# Warm, water-depleted rocky exoplanets with surface ionic liquids: A proposed class for planetary habitability

**DOI:** 10.1073/pnas.2425520122

**Published:** 2025-08-11

**Authors:** Rachana Agrawal, Sara Seager, Iaroslav Iakubivskyi, Weston P. Buchanan, Ana Glidden, Maxwell D. Seager, William Bains, Jingcheng Huang, Janusz J. Petkowski

**Affiliations:** ^a^Department of Earth, Atmospheric and Planetary Sciences, Massachusetts Institute of Technology, Cambridge, MA 02139; ^b^Department of Physics, Massachusetts Institute of Technology, Cambridge, MA 02139; ^c^Department of Aeronautical and Astronautical Engineering, Massachusetts Institute of Technology, Cambridge, MA 02139; ^d^Kavli Institute for Astrophysics and Space Research, Massachusetts Institute of Technology, Cambridge, MA 02139; ^e^Tartu Observatory, University of Tartu, Tõravere 61602, Estonia; ^f^Department of Chemistry and Biochemistry, Worcester Polytechnic Institute, Worcester, MA 01609; ^g^School of Physics and Astronomy, Cardiff University, Cardiff CF24 3AA, United Kingdom; ^h^Faculty of Environmental Engineering, Wroclaw University of Science and Technology, Wroclaw 50-370, Poland; ^i^JJ Scientific Mazowieckie, Warsaw 02-792, Polan

**Keywords:** exoplanets, ionic liquids, astrobiology

## Abstract

The discovery of thousands of exoplanets and the emergence of telescopes capable of exoplanet atmospheric characterization have intensified the search for habitable worlds. Due to selection biases, many exoplanets under study are planets deemed inhospitable because their surfaces are too warm to support liquid water. We propose that such planets could still support life through ionic liquids: Liquid salts with negligible vapor pressure that can persist on warm planets with thin atmospheres, where liquid water cannot. Ionic liquids have not previously been considered as naturally occurring substances, and thus have not been discussed in planetary science. We demonstrate in laboratory experiments that ionic liquids can form from planetary materials: Sulfuric acid combined with nitrogen-containing organic molecules. Sulfuric acid can be volcanic in origin, and organic compounds are commonly found on planetary bodies. The required planetary surface is water-depleted and must support sulfuric acid transiently in liquid phase to dissolve organics, followed by evaporation of excess liquid—conditions spanning approximately 300 K at 10^−7^ atm to 350—470 K at 0.01 atm. Because ionic liquids have extremely low vapor pressures, they are not prone to evaporation, allowing small droplets or pools to persist without ocean-like reservoirs. Ionic liquids’ minuscule vapor pressure at room temperature suggests possible stability on planets with negligible atmospheres, shielded by magnetic fields or rock crevices against harsh cosmic radiation. Ionic liquids can stably dissolve enzymes and other biomolecules, enabling biocatalysis and offering a plausible solvent for life—broadening the definition of habitable worlds.

## Significance

The search for habitable exoplanets has intensified with new telescopes and a growing number of exoplanets. Yet, many known exoplanets are too warm for surface liquid water and therefore considered inhospitable to life. Liquid is a fundamental requirement for life as we understand it, but whether that liquid has to be water is not known. We propose that such planets could still support life through ionic liquids, substances with negligible vapor pressure that remain liquid in warm, low-pressure conditions, even approaching a vacuum. Our experiments show that ionic liquids can form from planetary materials—sulfuric acid and nitrogen-containing organic compounds—offering a potential pathway for life on warm, thin-atmosphere, water-depleted worlds.

Humanity’s understanding of habitable worlds has expanded dramatically over the past few decades. We now know that several of Jupiter and Saturn’s icy moons, including Europa, show strong evidence of subsurface, salty global oceans (1), with the Europa Clipper (2) and Jupiter Icy Moons Explorer missions (3) now en route to explore Europa further. Titan, the only solar system body other than Earth with persistent surface liquids, has methane and ethane lakes. The Dragonfly mission is currently under development to explore Titan’s surface and atmosphere (4). Additionally, Venus has liquid droplets of sulfuric acid in its cloud layers, and new laboratory research shows that a subset of biologically relevant molecules remain stable in concentrated sulfuric acid, opening new doors to considering life in the temperate layers of the Venus atmosphere (5–12). With thousands of exoplanets now known, we have a growing array of possible habitable worlds, given their incredible diversity in mass, radius, and orbit. Our ability to fully observe and characterize terrestrial exoplanets with Earth-like conditions is still limited by current technology, motivating us to explore nontraditional pathways to habitability.

The model of terrestrial life, based on liquid water is so well established that considerations of habitability have been reserved largely for those rocky exoplanets that could have large stable surface reservoirs of liquid water and a supporting atmosphere. However, the diversity of environments in our solar system and the growing catalog of exoplanets inspire us to consider life in settings very different from Earth’s. Therefore, we adopt the more general set of life’s base requirements: a liquid solvent, temperatures suitable for covalent bonds (so complex molecules can form), and an energy source (13).

Under these general requirements, we propose a category of habitable planets—inspired by the observation that ionic liquids remain liquid even in vacuum at room temperature and can form from common planetary chemicals. Ionic liquids enable warm, rocky super Earths without surface water and thin atmospheres to have surface liquid, and hence potential to be habitable.

Ionic liquids are salts in a liquid state, typically composed of ions rather than neutral molecules. They can remain liquid at a wide range of temperatures and pressures, including conditions where water would evaporate or freeze. Ionic liquids are formally defined as salts with melting temperatures below 100 °C (14). Ionic liquids differ from conventional liquids in that they typically lack a triple point. Rather than boiling, they decompose at the gas-phase boundary, and they often crystallize at temperatures above their melting points. Ionic liquid phase behavior is thus governed by solid–liquid equilibria and, in some cases, glass transitions, rather than solid–liquid–gas coexistence. Ionic liquids differ from each other in their characteristics (decomposition temperature, viscosity, etc.) but all share the property of very low vapor pressures. For example, many ionic liquids have vapor pressures as low as 10^−15^ bar at 300 K, remaining effectively non-volatile even under vacuum conditions (15).

There are thousands of known ionic liquids, with millions likely awaiting discovery (e.g., refs. 16 and 17). Ionic liquids have a wide range of applications, including electrochemical (e.g., in batteries), industrial catalysis, biopolymer processing, and pharmacology (18). Ionic liquids are synthetic^†^ and have not previously been considered as naturally occurring substances and so have not been discussed in the context of planetary science.

## 1. Results

**Fig. 1.**
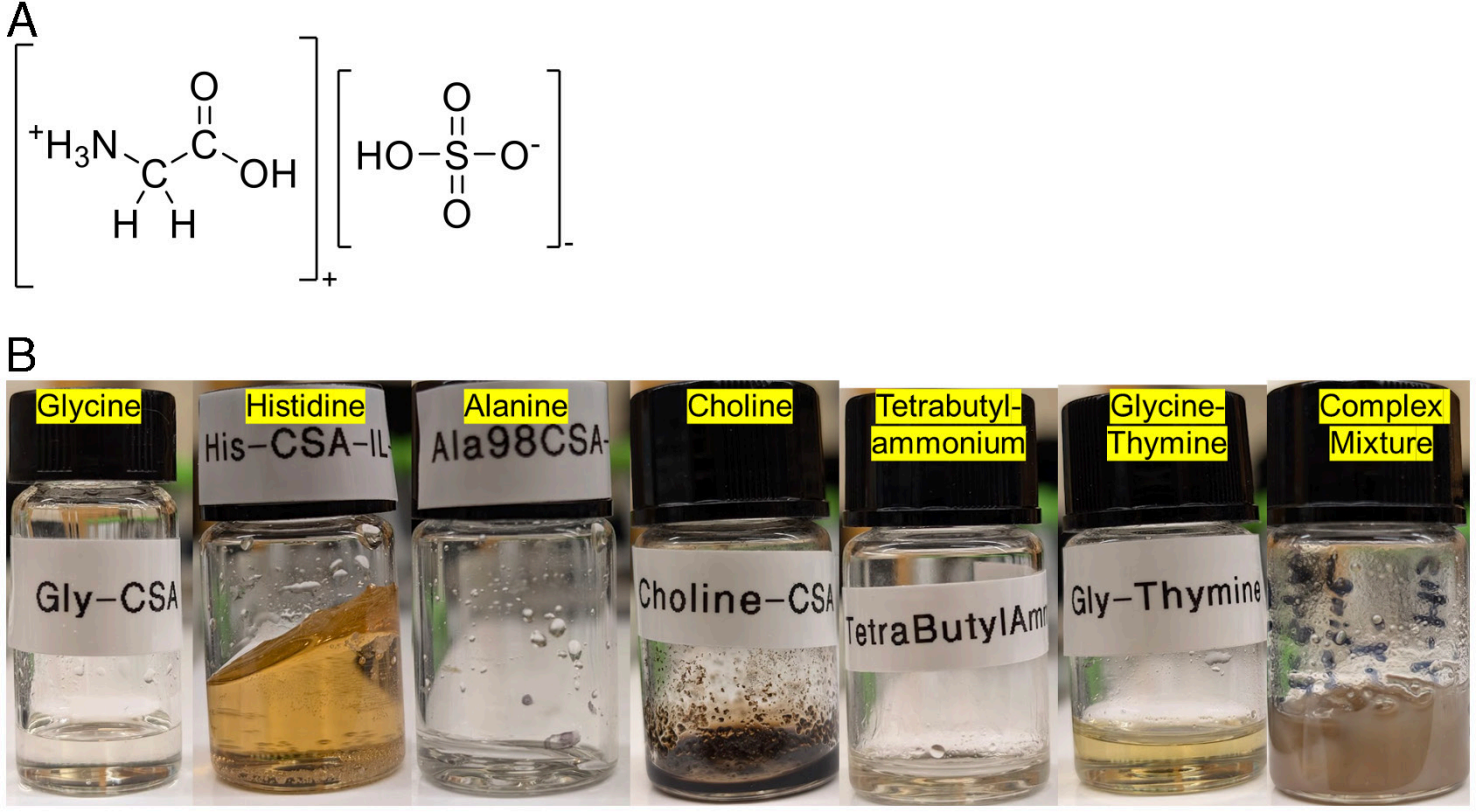
Ionic liquids. (*A*) schematic of an ionic liquid composed of a hydrogen sulfate anion and a glycine cation. (*B*) Vials of selected ionic liquids composed of N-containing organics and hydrogen sulfate anions, compounds as labeled in the image. Ionic liquids are typically transparent, with a range of chemical characteristics including colors and viscosities. The ionic liquids were generated by evaporating excess sulfuric acid from mixtures of various organic compounds and concentrated sulfuric acid. See *Materials and Methods* and *SI Appendix* for details.

We have found that ionic liquids form from planetary materials: concentrated sulfuric acid combined with a wide variety of nitrogen-containing organic molecules (Fig. 1) under a range of temperature–pressure conditions (*Materials and Methods*). The ionic liquid is composed of a hydrogen sulfate (HSO_4_^−^) anionic component and a protonated nitrogen-containing organic compound cationic component (19) (hydrogen sulfate ionic liquids; Fig. 1). In terms of planetary materials, the HSO_4_^-^ anion comes from sulfuric acid and the cationic component originates from organics found in meteorites, asteroid surfaces, and elsewhere, and also considered in prebiotic chemistry scenarios (20).

We form ionic liquids by first dissolving organic compounds in concentrated sulfuric acid followed by evaporation of the excess concentrated sulfuric acid under low pressure [at 10^−5^ bar and warm temperatures (100+/−5°C)]. For the evaporation procedure, we use a custom-built in-house vacuum chamber. Other combinations of temperatures and pressures also form ionic liquids (see *Materials and Methods* and *SI Appendix*, section S2).

We find ionic liquid formation for a wide variety of organic molecules that contain a nitrogen atom, including amino acids, aliphatic amines, nucleic acid bases, and other aromatic heterocycles. Once dissolved in concentrated sulfuric acid the N-containing organic molecules become protonated and gain a stable positive charge. This means that any nitrogen-containing organic molecule that is stable and soluble in concentrated sulfuric acid could form ionic liquids upon evaporation of the excess sulfuric acid.

We highlight ionic liquids with glycine, histidine, alanine, choline, tetrabutylammonium, as well as a mixture of glycine and thymine, and also a mixture of glycine, thymine, stearic acid, paraffin, and naphthalene (Fig. 1).

We find that as long as the positively charged organics are present, ionic liquids can also form from complex mixtures of diverse compounds, including hydrocarbons, carboxylic acids, sugars (saccharose), and other chemicals (*SI Appendix*, Table S1). The mixture of compounds indicates that impure, reactive, and highly complex solutions of organics do not prevent the formation of ionic liquids (*SI Appendix*, section S3 and Fig. S8). Furthermore, the concentration of sulfuric acid (i.e., dilution in water); the concentration of organics in sulfuric acid; and the overall volume of the initial organics and concentrated sulfuric acid mixture (down to the nL volume we could measure) do not inhibit the formation of ionic liquid (*SI Appendix*, section S4).

While we do not expect ionic liquids to be pure substances (i.e., formed from a single type of organic molecule) here we largely explore individual ionic liquids for proper chemical characterization, while also including two example mixtures.

**Fig. 2.**
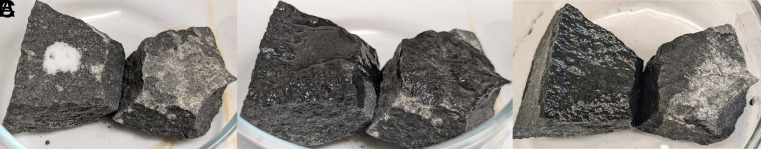
[glycine^+^][HSO_4_^−^] ionic liquid formation on a basaltic rock. (*A*) *Left* rock: Glycine powder on the surface of the basalt rock. *Right* rock: No glycine added on the surface of basalt rock. (*B*) Equal amounts of hot 98% w/w H_2_SO_4_ (the rest water) poured over both rocks [same rocks as in (*A* and *C*)]. (*C*) Basaltic rocks after 24 h under 10^−5^ bar pressure and 80 +/−5 °C. Left rock: Formation of stable [glycine^+^][HSO_4_^−^] ionic liquid on the surface of basalt. Right rock: In contrast, without the presence of glycine 98% w/w sulfuric acid evaporates completely.

For more realistic planetary-like conditions, we used basaltic rock as a platform to form ionic liquids. We used a mixture of N-containing organics and concentrated sulfuric acid on the surface of basaltic rocks, under a variety of conditions (80 °C and 10^−5^ bar, as well as room temperature, room pressure both in dry air and room humidity). The ionic liquids that form on the surface of basaltic rocks are stable to further reactivity (Fig. 2 and *SI Appendix*, section S4 and Figs. S18 and S19).

We support the composition of the HSO_4_^−^ ionic liquids with Fourier transform infrared spectroscopy (FTIR) and ^1^H NMR spectroscopy. We use [glycine^+^] [HSO_4_^−^] as our main example with several others detailed in the *SI Appendix*, Table S1 and section S3).

**Fig. 3.**
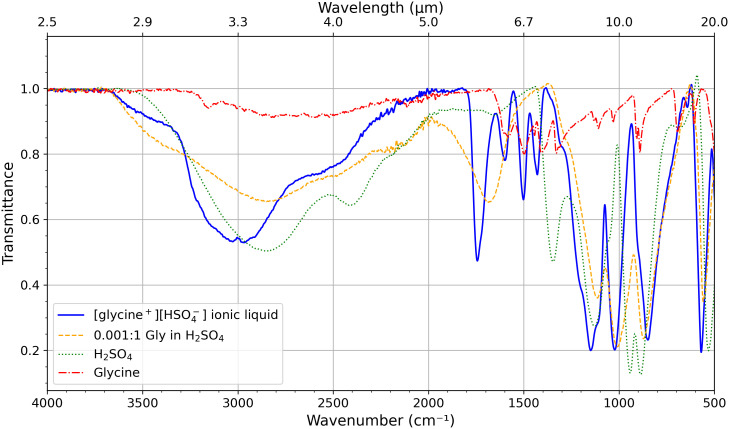
FTIR spectra of [glycine^+^][HSO_4_^−^] ionic liquid and components. The *y*-axis is transmittance and the *x*-axis is wavenumber in cm^−1^. The spectrum of [glycine^+^][HSO_4_^−^] ionic liquid is distinct from the others, namely pure glycine, pure 98% w/w H_2_SO_4_ (the rest water), and glycine dissolved in 98% w/w H_2_SO_4_, and furthermore is consistent with the spectra of other hydrogen sulfate ionic liquids reported in the literature (e.g., ref. 19). See also *SI Appendix*, Table S5, Figs. S5–S9 and S4.

FTIR (Fig. 3). For all FTIR-measured ionic liquids, we detect the ν_S=O_ and ν_S-O_ features of HSO_4_^−^(*SI Appendix*, Table S5). We show that the [glycine^+^] [HSO_4_^−^] ionic liquid differs from Gly dissolved in concentrated sulfuric acid as well as from the individual Gly and H_2_SO_4_ components. The pure H_2_SO_4_ has a prominent peak at 1345 cm^−1^, corresponding to SO-H bending vibrations. These vibrations are not prominent in the ionic liquid due to the strong ionic bond. Our FTIR spectrum is also consistent with previously reported FTIR spectra of HSO_4_^−^ ionic liquids (19).

**Fig. 4.**
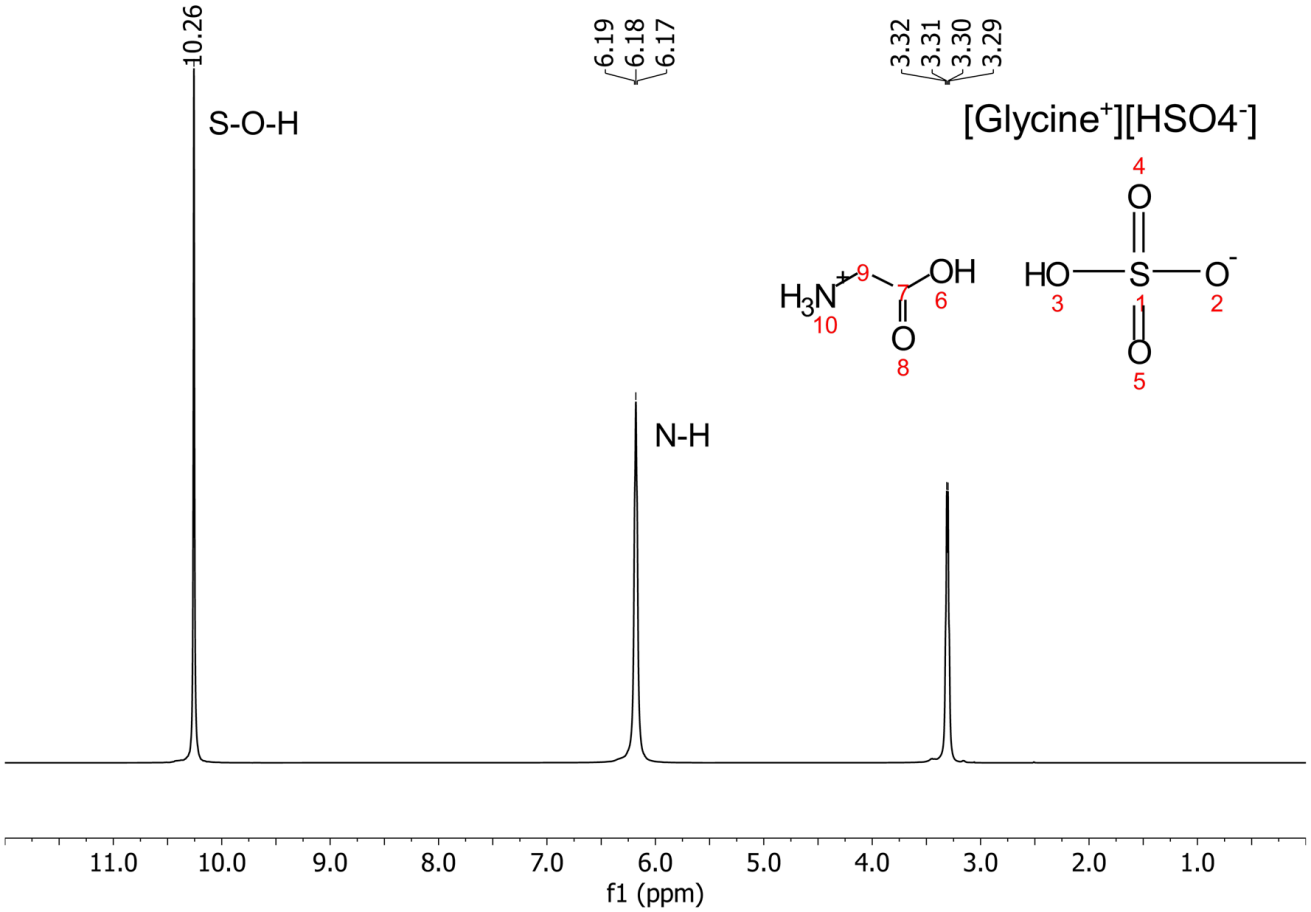
^1^H NMR spectra of [glycine^+^][HSO_4_^−^] ionic liquid. The spectra were recorded at 80 °C and 500.18 MHz with DMSO-d_6_ as the external lock solvent. The peak at 10.26 ppm, corresponds to the SO-H proton of the [HSO_4_^−^] anion while the peak at 6.18 ppm corresponds to the amine hydrogens of the protonated glycine molecule, confirming the identity of the ionic liquid. Additionally, the peak at 3.3 ppm is from the H from the CH_2_ group of glycine, and no other peaks are visible, confirming the overall chemical composition of the sample.

^1^H NMR (Fig. 4). For all NMR-measured ionic liquids, we identify two sets of protons, in the 6 to 12 ppm region of the ^1^H NMR spectrum, that are characteristic of cationic and anionic species. For the [glycine^+^][HSO_4_^−^] ionic liquid, the most downfield-shifted peak, at 10.26 ppm, corresponds to the SO-H proton of [HSO_4_^−^] anion, while the peak at 6.18 ppm corresponds to the amine hydrogens of the protonated glycine molecule (*SI Appendix*, section S3 and Fig. S4).

We also determined the acidity of selected ionic liquids and find that they are lower than concentrated sulfuric acid but still acidic, with the Gutmann-Beckett acceptor number (AN) values ranging from 117 to 98, as compared to H_2_SO_4_ of 122 (*SI Appendix*, section S3 and Table S6). Our measured AN values are consistent with those reported for other hydrogen sulfate ionic liquids (19). The AN is a quantitative measure of acidity of the solution, determined by the ^31^P NMR chemical shift of triethylphosphine oxide (TEPO) when interacting with acid molecules in solution (21, 22). Higher AN values indicate higher acidity of the solution as compared to lower AN values.

We have implicitly described our ionic liquids as equimolar, expected because of the evaporation of excess sulfuric acid under 80 to 100 °C and low-pressure conditions. To support the equimolar nature of our [glycine^+^][HSO_4_^−^] ionic liquid, we prepared an equimolar ionic liquid directly, by adding equal molar amounts of glycine and H_2_SO_4_ (i.e., without the evaporation step) and found a similar AN value (112.8) to the [glycine^+^][HSO_4_^−^] ionic liquid formed with excess H_2_SO_4_ evaporation (113.1).

The ionic liquids have various decomposition temperatures depending on molecules and mixtures, but all higher than about 180 to 190 °C (Table 1). Also relevant is that ionic liquids are highly hygroscopic.

**Table 1.** Thermal decomposition temperatures of selected ionic liquids

No.	Sample	T_0_(°C)	T_50_(°C)
1	[glycine^+^][HSO_4_^−^]	199.44	222.66
2	[cysteine^+^][HSO_4_^−^]	185.91	205.98
3	[histidine^+^][HSO_4_^−^]	180.74	281.28
4	[valine^+^][HSO_4_^−^]	191.4	212.58
5	[thymine^+^][HSO_4_^−^]	248.59	299.85
**6**	[guanine^+^][HSO_4_^−^]	225.3	232.27
7	[purine^+^][HSO_4_^−^]	229.86	297.77
8	[diaminopurine^+^][HSO_4_^−^]	202.24	296.31
9	[tetrabutylammonium^+^][HSO_4_^−^]	270.78	285.99
10	[1-ethyl-3-methylimidazolium^+^][HSO_4_^−^]	345.65	379.91
11	[choline^+^][HSO_4_^−^]	252.54	290.71

T_0_ is the temperature of onset of decomposition and T_50_ is the temperature at which 50% weight is lost.

## 2. Discussion

### 2.1. Formation of Ionic Liquids in a Planetary Environment.

Ionic liquids can conceivably form on a rocky exoplanet when sulfuric acid comes into contact with nitrogen-bearing organic compounds, dissolving them in an environment where excess water and sulfuric acid can evaporate.

#### 2.1.1. Sulfuric acid.

For hydrogen sulfate ionic liquids to form, a planet must have a source of liquid sulfuric acid. The core production mechanism is the general reaction SO_2_ + H_2_O $\overset{\left[O\right]}{\to }$ H_2_SO_4_ via oxidation—a universal pathway likely to occur on any planet with the necessary reactants. On volcanically active planets, SO_2_ and H_2_O vapor can be exsolved from the mantle. The critical step in H_2_SO_4_ formation is the oxidation of SO_2_ to SO_3_, which requires reactive oxygen species (O) such as O, O_2_, O_3_, or H_2_O_2_ to overcome the high activation energy barrier (23). Industrial production of sulfuric acid similarly relies on reactive oxygen and a catalyst to drive this step. On planets, the required oxygen can be supplied by UV photodissociation of oxygen-bearing molecules like CO_2_ (also volcanically exsolved), H_2_O, and others. For example, on Venus, UV-driven oxidation of volcanic SO_2_ to SO_3_, followed by reaction with trace atmospheric water, produces the planet’s dense sulfuric acid clouds (24). An analogous process occurs in Earth’s stratosphere, where photochemically generated OH oxidizes SO_2_ to form H_2_SO_4_ (25). Sulfuric acid may also form at the surface when SO_2_ reacts with basaltic or mafic rocks. On Earth, Fe_2_O_3_-bearing rocks catalyze this reaction in SO_2_-rich volcanic environments, such as at Kīlauea, Hawai’i (26). Atmospheric O_2_ likely replenishes lattice oxygen in the Fe_2_O_3_ catalyst; a similar mechanism could operate on exoplanets without O_2_, though this remains to be investigated (e.g., volcanic NO_2_, such that SO_2_ + NO_2_ → SO_3_ + NO for an overall SO_2_ + 2NO_2_ + H_2_O → H_2_SO_4_ + 2NO). Sulfuric acid has also been observed on Europa, where it forms through radiolytic oxidation of surface sulfur (reviewed by ref. 23). In summary, volcanically exsolved SO_2_ and H_2_O can generate sulfuric acid through diverse pathways, and concentrated H_2_SO_4_can condense even under low atmospheric pressure.

The availability of hydrogen may initially seem problematic on a water-depleted planet. However, Venus serves as a counter example: despite limited hydrogen, it hosts abundant sulfuric acid. On Venus, both SO_2_ and H_2_O are outgassed from volcanoes, and UV radiation drives their conversion to H_2_SO_4_. The H_2_O/SO_2_ ratio in Venusian volcanic gases is relevant but not well constrained. Zolotov et al. (27) report that SO_2_ dominates the molar fraction over water, estimating SO_2_ could approach 10%, with CO_2_ making up the remaining 90%. They note that water is not considered an important volcanic gas on Venus. Modeling by Constantinou et al. (28) places an upper limit of 6% for the water mole fraction. By contrast, Earth’s volcanic emissions are ~90% water by mole fraction, making H_2_O the dominant gas (29). These comparisons underscore that sulfuric acid can plausibly form even in hydrogen-limited environments. The work of Janssen et al. (30) supports the argument that H_2_SO_4_ is likely to exist in sulfur-rich, oxygen-rich, and hydrogen-poor planetary conditions. See *SI Appendix*, Table S7.

**Fig. 5.**
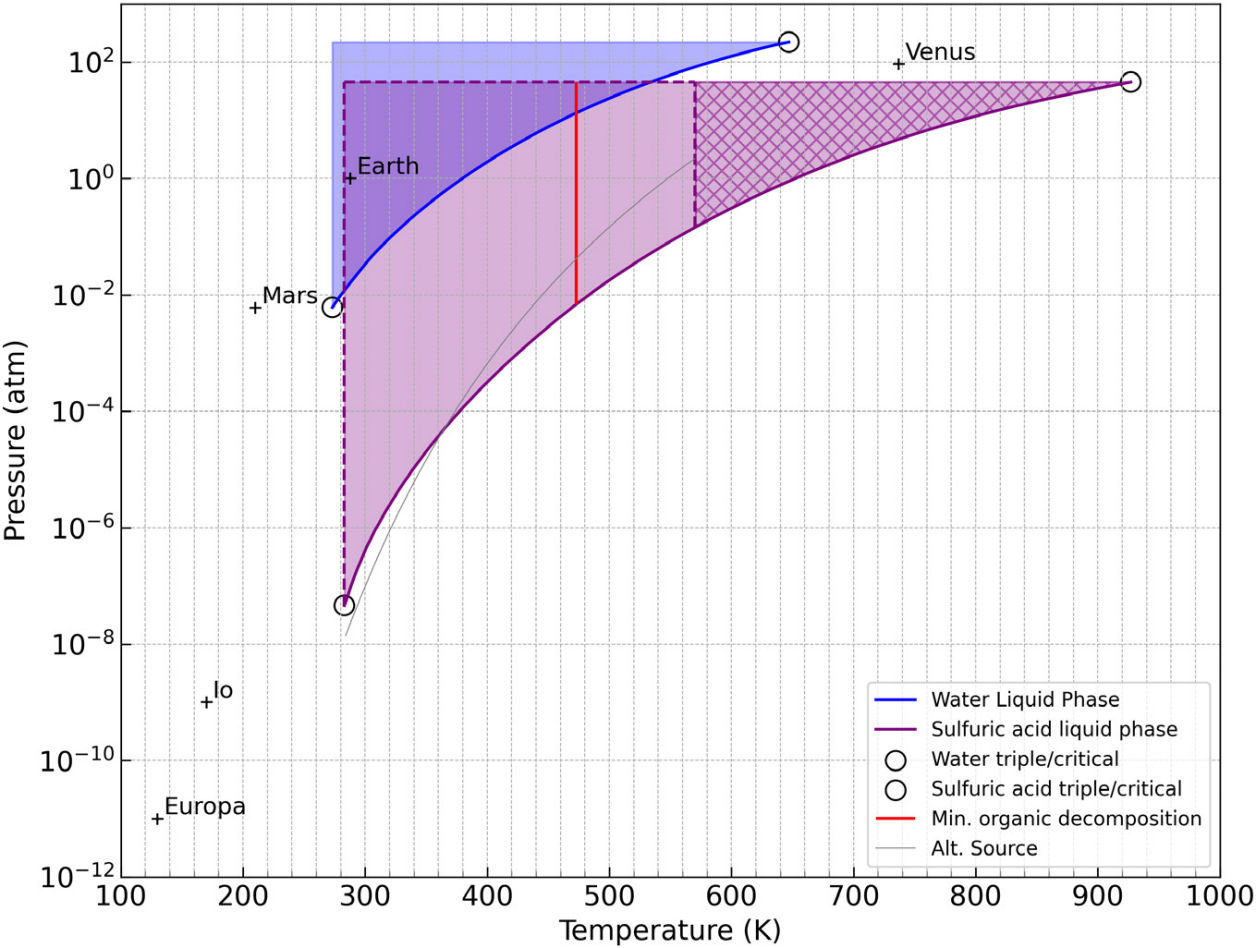
Sulfuric acid phase diagram. The pressure in atm (*y*-axis) vs. temperature in K (*x*-axis). The large purple shaded region represents the theoretical liquid-phase boundary for 98% w/w concentrated sulfuric acid (the rest water), adapted from Ballesteros et al. (31), based on their compiled experimental data from ref. 34. Our light gray curve fit follows the data from (32), and diverges from the (31) below 475 K due to different thermodynamic sources. Above about 570 K, H_2_SO_4_ decomposes into SO_3_ and H_2_O rather than existing as a stable gas-phase species, shown by the hatched purple region. The solid red line marks an approximate thermal threshold above which organic compounds are expected to thermally break down, where ~470 K is conservative from the lower range of our measured decomposition temperatures (Table 1). The blue shaded region indicates the liquid phase of water. The region to the left of the purple and red lines [and spanning about 100 K colder than the purple line (*SI Appendix*, Table S9)] includes conditions under which planet surfaces may allow liquid H_2_SO_4_ to persist, dissolve organic material, and evaporate, leading to the formation of hydrogen sulfate ionic liquids.

H_2_SO_4_ is a liquid over a wide range of temperature and pressure conditions. The key question for ionic liquid formation is whether liquid sulfuric acid can exist at the planetary surface long enough to dissolve organic compounds, then evaporate efficiently to leave behind an ionic liquid. The answer is yes—formation and evaporation is viable across a broad range: from low pressures and temperatures (~300 K at 10^−6^ atm) to higher pressures and temperatures (~360 to 470 K at 10^−^² atm, pressures comparable to Mars-like conditions). We adopt 470 K as an upper temperature cutoff, not because sulfuric acid cannot exist as a liquid above this temperature, but because many of the ionic liquids we study begin to degrade beyond that point (Table 1). A phase diagram for sulfuric acid with estimated boiling curves is shown in Fig. 5, adapted from ref. 31 and supplemented by our own estimated boiling points from the vapor pressure–temperature relationship via the Antoine equation and data from ref. 32 and 33. For example, we estimate that concentrated sulfuric acid can evaporate within a month (a subjective choice) within the range of about 100 K cooler than the boiling point, based on our experimental evaporation measurements (*SI Appendix*, Table S9).

Although we favor the warm, thin atmosphere exoplanet as a host to hydrogen sulfate ionic liquids, our extended experiments on basalt rocks (*SI Appendix*, Fig. S19) show that even at room temperature, room pressure, room humidity conditions ionic liquid can form once the sulfuric acid dissolves the N-containing organic—as long as the excess sulfuric acid disperses by evaporation, spreading into rock pores, reaction with the rock, etc.

#### 2.1.2. Surface organics.

For an ionic liquid to form, concentrated sulfuric acid must dissolve nitrogen-containing organics. The planet must therefore have pockets of organic material on its surface. So far, organics have been detected on every solid-surfaced body where they have been specifically searched for (Mercury, the Moon, Mars, Ceres, Ganymede and Callisto, Enceladus, Titan, Pluto and Charon, comets and asteroids). How these organics formed—and their chemical character—remains an outstanding question in planetary science. For details and relevance to ionic liquid formation, see *SI Appendix*, Table S10.

On the vast majority of these bodies, organics’ concentrations are not measured; however, detection itself implies substantial local deposits. For example, on Mercury, a ~10 cm deep layer of organics covers the water ice deposits in the polar craters (35). Comet 67P has an organic carbon content of ~45 wt% in dust particles ranging from 50 to 1,000 μm in size (36), showing that at the microscale, organic matter can be highly concentrated. While the spatial extent and exact composition of organics on these cometary particles remains unmeasured (e.g., surface coverage, clumping, N-containing percentage, etc.), their presence confirms the existence of dense organic deposits. On meteorites, organic matter can occur in globular deposits with variable sizes, typically ranging from 50 to 300 nm, and is often highly concentrated (37).

In our own solar system no bodies are known to have ionic liquids. Io does not have liquid sulfuric acid, likely both because it is severely H-depleted and too cold for liquid sulfuric acid. Mercury does not have any active volcanoes, so current formation of H_2_SO_4_ is not possible. Notably, Europa has solid sulfuric acid on its surface, showing that H_2_SO_4_ does not sublimate even at 10^−12^ atm under frigid temperatures.

### 2.2. Atmosphereless Worlds.

The range of exoplanet surface conditions may extend down to atmosphereless worlds, enabled by the extremely low vapor pressure of ionic liquids. Even a small droplet of many ionic liquids will not “dry out,” even under vacuum at Earth-like temperatures (38). This makes the persistence of ionic liquids plausible on surfaces lacking atmospheric pressure. However, exposure to the harsh space environment could lead to space weathering of the surface material. Phenomena such as micrometeorite impacts, solar wind plasma, UV, and high-energy radiation could alter the chemical and physical properties of the unprotected planetary surface, including any surface ionic liquid (39, 40). The penetration depth of space weathering ranges from 2 to 3 topmost atomic layers, due to ion sputtering, to 100 nm for extremely high-energy protons. On the other hand, the depth of penetration of galactic cosmic rays with GeV energy is much larger than high-energy protons and can reach 1 m (39). Thus, any subsurface ionic liquid, even in shallow depths, could be protected from most harsh space weathering processes. A protective magnetic field could help shield the surface, and additional protection could come from subsurface environments, shaded regions, or crevices where ionic liquids can be shielded from direct exposure. For example, ionic liquids might survive on the dark side of a tidally locked planet near warm volcanic vents, assuming the planet maintains a magnetic field. The long-term survival of ionic liquids—particularly their resistance to UV and energetic particle bombardment—is an open question and a topic for future study.

### 2.3. A Range of Ionic Liquids.

Hydrogen sulfate ionic liquids might just be one example out of many possible planetary ionic liquids. In principle any positively charged organic molecule could act as a cation in an ionic liquid pair. There are also at least several possible anions that could pair with organic cations, such as Cl^−^, NO_3_^−^, ClO_4_^−^, HCOO^−^, CH_3_COO^−^, and deep eutectic solvents which are related to ionic liquids but do not have charged components. Indeed, none of the planetary bodies in our own solar system are expected to have surface ionic liquids because they are either too cold for the liquid phase or lack the necessary chemical components.

### 2.4. Habitability.

We now turn to support our title statement “habitable world”. The building blocks of our water-based biochemistry, such as DNA and proteins (enzymes) are often stable and functional in the presence of ionic liquids (reviewed in refs. 41, 42), and some even function in dehydrated pure ionic liquids (e.g., reviewed in ref. 43). Finally, many ionic liquids are not toxic (44) and are chemically benign and unreactive when in contact with Earth life biochemicals (e.g., ref. 43).

Ionic liquid cationic components like quaternary amines and other charged N-containing organic compounds are readily made by life and the anionic components of ionic liquids, such as H_2_SO_4_, can be present in the right planetary environment. Ionic liquids are polar and can dissolve salts and complex polymers, which meets some of the criteria suitable for a solvent for life (13). Many species use or produce compounds that could be used to make ionic liquids, such as amines or sugars, as a means to survive desiccation (e.g., ref. 45). Moreover, because ionic liquids do not evaporate the risk of permanent desiccation for an organism using an ionic liquid as a solvent does not exist, leaving only the risk of mechanical damage as a cause of solvent loss to the outside world.

A planet may initially have only small isolated pockets of ionic liquid, due to the limiting amount of input ingredients, sulfuric acid, and organics. Therefore, the ionic liquid is likely itself not a dominant planetary surface feature, meaning it is likely currently undetectable. If there are large reservoirs of surface ionic liquid, future telescopes that can spatially resolve the surface during secondary eclipse ingress and egress may be able to detect the sulfate group via the planet’s emission spectra. If life permeates the surface, perhaps its pigments would show a feature in reflected light (e.g., vegetation’s “red edge”). Life itself might generate waste gases from exploiting chemical redox gradients in the environment. Any gas would diffuse upward and get photodissociated, with atoms escaping the planet. It is possible that a powerful UV telescope is able to spot such an exosphere with unusual ratios of atoms that could be further explored as a sign of life.

In summary, ionic liquids can exist as stable liquids under surface conditions that are too warm or too low in pressure for liquid water to persist. This includes warm planets with thin atmospheres and possibly even atmosphereless bodies, where the extremely low vapor pressure of ionic liquids may prevent them from evaporating if protected from UV and harsh cosmic radiation. Liquid is a fundamental requirement for life as we understand it (46), and the ability of ionic liquids to form and remain stable in these environments expands the range of planetary conditions where life could potentially arise. Ionic liquid formation from volcanic sulfuric acid and surface organics—both observed in the laboratory and now expected on some rocky bodies—introduces a new class of potentially liquid-bearing planets. This significantly broadens the concept of planetary habitability beyond the traditional water-based paradigm.

## 3. Materials and Methods

### 3.1. Materials.

We purchased sulfuric acid (95 to 98%), glycine (Cat. No. G7126), histidine (Cat. No. H8000), thymine (Cat. No. T0376), adenine (Cat. No. A8626), purine (Cat. No. P55805), guanine (Cat. No. G11950), diaminopurine (Cat. No. 247847), tetrabutylammonium hydroxide (Cat. No. 178780), acetic acid (Cat. No. 695092), oxalic acid (Cat. No. 241172), benzoic acid (Cat. No. 242381), succinic acid (Cat. No. S9512), stearic acid (Cat. No. S4751), palmitic acid (Cat. No. P0500), and dimethyl sulfoxide-d_6_ (1034240100) from Sigma-Aldrich. The remaining amino acids—alanine, arginine, asparagine, aspartic acid, cystine, lysine, methionine, tryptophan, valine, glutamine, glutamic acid, isoleucine, leucine, phenylalanine—were part of a kit of 20 amino acids from Sigma-Aldrich (Cat. No. LAA21-1KT). Choline hydroxide (Cat. No. 224210) was purchased from Beantown Chemicals and 1-Ethyl-3-methylimidazolium hydrogen sulfate (H27232) and pyrimidine (157700050) from Thermo Scientific Chemicals. The triethylphosphine oxide (Cat. No. AA3039103) was purchased from Fisher Scientific. Alanylglycine dipeptide (Cat. No. ALA121374-5G) and basalt rock samples Ward’s Basalt (Cat. No. 470025-914) were purchased from VWR.

### 3.2. Preparation of Ionic Liquids.

To form equimolar ionic liquids, we mixed approximately equal moles of the organic compound and 98% w/w concentrated sulfuric acid (the rest water). We then added about 5% extra H_2_SO_4_ to ensure the organic completely dissolved in sulfuric acid. To further aid solubility, the mixture was incubated in a sand bath at 80+/−5 °C for 12 to 24 h. The time required to dissolve varies depending on the organic molecule. Once the organics are completely dissolved, we evaporated the excess sulfuric acid from the mixture inside a custom-made low-pressure evaporator system (100+/−5 °C and 10^−5^ bar pressure). These temperature–pressure conditions allow excess sulfuric acid to evaporate slowly. More details on quantities and evaporation time are provided in *SI Appendix*, section 2. The remaining substance is a highly viscous ionic liquid. We followed the same procedure in the preparation of all other ionic liquids from all other nitrogen-containing compounds.

Here, we have described our method for forming ionic liquids, we emphasize that the initial concentrations of organics in sulfuric acid, the exact evaporation temperatures and pressures, and the timescales can be widened. What matters is the complete evaporation of excess concentrated sulfuric acid (which depends on temperature and pressure, see *SI Appendix*, section S2 and Table S9). We emphasize that equimolar ionic liquids can form even at room temperature and pressure, in the situation where there is no excess sulfuric acid to be evaporated. In the lab, we optimize for efficiency by using excess sulfuric acid to aid dissolution of the organic (typically in powder form) and evaporating at our pumping system’s minimum pressure, but the underlying conclusion remains: Initial concentrations are not critical.

### 3.3. ^1^H NMR Spectroscopy.

We used ^1^H NMR spectra to confirm the structure of selected ionic liquids. The ionic liquids were transferred to NMR tubes (5 mm, borosilicate glass). A sealed capillary tube containing a deuterated solvent dimethyl sulfoxide-d_6_ (DMSO-d_6_), an external lock solvent, was inserted into the NMR tube containing the ionic liquid. We obtained the ^1^H NMR spectra at 80 °C using a Bruker Avance Neo spectrometer operating at 500.18 MHz.

### 3.4. Acceptor Number Determination.

We used the Gutmann Beckett Acceptor Number method to determine the Lewis acidity of selected ionic liquids (21, 22). We used triethylphosphine oxide (TEPO) as the probe for ^31^P NMR. We added approximately 50 mg of TEPO to ~1 mL of the ionic liquid and incubated the sample at 80 °C to allow mixing. The samples were transferred into 5 mm NMR tubes with a capillary tube insert containing DMSO-d_6_ as an external lock. ^31^P NMR spectra were obtained at 80 °C using a Bruker Avance Neo spectrometer operating at 500.18 MHz. We measured the spectra for H_2_O and H_2_SO_4_ to establish the range of acidity within which the ionic liquid acidity must lie.

### 3.5. FTIR Spectroscopy.

Infrared spectra of ionic liquids, the organics, and H_2_SO_4_ were measured using the Bruker Alpha II FTIR spectrometer with a Diamond Crystal ATR (Attenuated Total internal Reflectance) accessory. We acquired 64 scans for each sample with 2 cm^−1^ resolution. See *SI Appendix*, section 2 for details.

### 3.6. Thermogravimetric Analysis (TGA).

We used the TGA method to determine the thermal decomposition onset temperature of selected ionic liquids. About 1 to 2 mg of ionic liquid was loaded on a platinum pan. The samples were heated from 30 °C to 600 °C with a rate of 10 °C/min under the flow of nitrogen. The weight change was measured using TA Instruments Discovery TGA 5500.

## Supplementary Material

Appendix 01 (PDF)

## Data Availability

The original data files have been deposited in Zenodo (47).

## Footnotes

^*^The only known example of the natural formation of ionic liquids on Earth is a result of detoxification of a venom of the fire ant (*Solenopsis invicta*) by a rival ant species, the tawny crazy ant (*Nylanderia fulva*) (48). The fire ant produces a deadly solenopsin insecticide containing a piperidine ring. After being sprayed with fire ant solenopsin venom, the tawny crazy ant can detoxify by grooming itself with its own venom, formic acid. The formic acid mixes with the *S. invicta* venom and protonates the N atoms of the piperidine rings. The resulting mixture is a viscous naturally produced ionic liquid that is no longer toxic to *N. fulva*.
