# Supplementary material for: Warm, water-depleted rocky exoplanets with surface ionic liquids: A proposed class for planetary habitability
Source: Proc Natl Acad Sci U S A. 2025 Aug 11;122(33):e2425520122. doi: 10.1073/pnas.2425520122 (PMC12377647; doi:10.1073/pnas.2425520122)
Supplement: Supplementary file 1 — Appendix 01 (PDF) [file pnas.2425520122.sapp.pdf]

PNAS

Supplementary Information for

Warm, Water-Depleted Rocky Exoplanets with Surface Ionic Liquids: A Proposed Class for Planetary Habitability

Rachana Agrawal[1], Sara Seager[1,2,3,4*], Iaroslav Iakubivskyi[1,5], Weston P. Buchanan[1], Ana Glidden[1,4], Maxwell D. Seager[6], William Bains[7], Jingcheng Huang[1], Janusz J. Petkowski[8,9,*]

[1] Department of Earth, Atmospheric and Planetary Sciences, Massachusetts Institute of Technology, 77 Massachusetts Avenue, Cambridge, MA 02139, USA
[2] Department of Physics, Massachusetts Institute of Technology, 77 Massachusetts Avenue, Cambridge, MA 02139, USA
[3] Department of Aeronautical and Astronautical Engineering, Massachusetts Institute of Technology, 77 Massachusetts Avenue, Cambridge, MA 02139, USA
[4] Kavli Institute for Astrophysics and Space Research, Massachusetts Institute of Technology, 77 Massachusetts Avenue, Cambridge, MA 02139, USA
[5] Tartu Observatory, University of Tartu, 1 Observatooriumi, 61602 Tõravere, Estonia
[6] Department of Chemistry and Biochemistry, Worcester Polytechnic Institute, Worcester, MA 01609, USA
[7] School of Physics and Astronomy, Cardiff University, 4 The Parade, Cardiff CF24 3AA, UK
[8] Faculty of Environmental Engineering, Wroclaw University of Science and Technology, 50-370 Wroclaw, Poland
[9] JJ Scientific, Warsaw, Mazowieckie, Poland

*Corresponding author: Sara Seager, Janusz J. Petkowski
Email: seager@mit.edu, janusz.petkowski@pwr.edu.pl

This PDF file includes:

Supplementary text
Figures S1 to S19
Tables S1 to S10
Supplementary References

Supplementary Information Text

S1. Ionic Liquids Summary

Ionic liquids are formally defined as salts with melting temperatures below 100 °C (1). Ionic liquids are synthetic and have not previously been considered as naturally occurring substances. We show that ionic liquids form with planetary materials: a wide range of N-containing organics as the cation, paired with a hydrogen sulfate anion (hydrogen sulfate salts). Here we summarize all the organics and mixtures we tried (Table S1). The 20 biogenic amino acids form ionic liquids at room temperature. The nucleic acid bases we tried also form hydrogen sulfate salts but were solid at room temperature; a subset were liquid at 100 °C, while others remained solid. We also show that mixtures of two or more organics with at least one N-containing organic also form ionic liquids.

Additionally, we tested six carboxylic acids (aliphatic and aromatic) as cations for ionic liquids. We hypothesized that molecules with a carbonyl group might also form ionic liquids with concentrated sulfuric acid (2), as the oxygen will gain a positive charge due to the protonation of the carbonyl oxygen (3–5). We found that they do not form ionic liquids under tested conditions.

We further characterize selected ionic liquids using a variety of analytical chemical techniques. We determine the composition, decomposition temperatures, and acidity by using ^{1}H Nuclear Magnetic Resonance (NMR) spectroscopy, Fourier transform infrared (FTIR) spectroscopy, Thermogravimetric Analysis (TGA), and ^{31}P NMR, respectively. More details of the experiments and analysis results are given in subsequent sections. Table S1 points towards the relevant sections for the selected ionic liquids.

To evaluate the plausibility of ionic liquid formation under diverse planetary conditions, we conducted a set of experiments examining chemical composition, environmental variability, and physical constraints. We varied the concentrations of both sulfuric acid and organics, assuming organics are the limiting component. We tested a range of organic concentrations diluted in concentrated sulfuric acid (Table S2), including as low as 20 mM glycine, which produced a similar FTIR spectrum to the equimolar mixture. Given the hygroscopic nature of ionic liquids, we also tested sulfuric acid diluted in water (0–95% w/w H_2SO_4, the rest water), and observed that, after evaporation of excess water and sulfuric acid, the resulting ionic liquid was qualitatively similar across this concentration range (Section S4). To examine whether ionic liquids can form at planetary-relevant microscale levels, we attempted to generate the smallest detectable quantities, constrained only by FTIR detection limits (Section S4). In addition, we expanded tests on rock surfaces (beyond Figure 2 and related discussion) to assess formation in more complex, non-pure environments (Section S3). While we conservatively set an upper temperature limit of 200 °C based on observed decomposition of the most labile compounds, this threshold likely varies; more thermally stable ionic liquids may persist at higher temperatures. Overall, our results highlight the robustness of ionic liquid formation across a wide range of starting conditions, supporting their plausibility on warm, water-depleted planetary surfaces.

Table S1. Summary of the hydrogen sulfate (HSO_4^-) ionic liquid formation for selected organic compounds. See Section S3 for more details on the characterization and analysis of selected ionic liquids. RT is room temperature.

No.	Organic Components	Ionic Liquid	Visual Observation	Analysis
**Amino Acids**				
1	Alanine	Yes	Clear, viscous	^{1}H NMR, FTIR
2	Arginine	Yes	Clear, viscous	Visual
3	Asparagine	Yes	Clear, viscous	Visual
4	Aspartic acid	Yes	Yellowish, viscous	Visual
5	Cysteine	Yes	Clear, viscous	^{1}H NMR, FTIR, TGA
6	Glutamine	Yes	Clear, viscous	Visual
7	Glutamic acid	Yes	Clear, viscous	Visual
8	Glycine	Yes	Clear, viscous	^{1}H NMR, FTIR, TGA, ^{31}P NMR
9	Histidine	Yes	Yellowish, highly viscous	FTIR, TGA
10	Isoleucine	Yes	Clear, viscous	Visual
11	Leucine	Yes	Clear, viscous	Visual
12	Lysine	Yes	Clear, viscous	Visual
13	Methionine	Yes	Red, viscous	Visual
14	Phenylalanine	Yes	Clear, viscous	Visual
15	Proline	Yes	Clear, viscous	Visual
16	Serine	Yes	Clear, viscous	Visual
17	Threonine	Yes	Clear, viscous	Visual
18	Tryptophan products	Yes	Dark, viscous	^{1}H NMR
19	Tyrosine	Yes	Yellowish, viscous	Visual
20	Valine	Yes	Clear, viscous	^{1}H NMR, FTIR, TGA
**Nucleic Acid Bases**				
21	Purine	Yes	Solid at RT, liquid at 100 °C	FTIR, TGA
22	Adenine	Yes	Solid at RT, liquid at 100 °C	Visual
23	Guanine	Yes	Solid at RT, liquid at 100 °C	FTIR, TGA
24	Diaminopurine	No	Solid at 100 °C	TGA
25	Pyrimidine	No	Solid at 100 °C	Visual
26	Thymine	Yes	Solid at RT, liquid at 100 °C	^{1}H NMR, FTIR, TGA
**Carboxylic Acids**				
27	Acetic acid	No	Completely evaporates	Visual

28	Benzoic acid	No	Completely evaporates	Visual
29	Oxalic acid	No	Completely evaporates	Visual
30	Succinic acid	No	Completely evaporates	Visual
31	Stearic acid	No	Completely evaporates	Visual
32	Palmitic acid	No	Completely evaporates	Visual
**Other N-containing Organics**				
33	Tetrabutylammonium	Yes	Clear	^{1}H NMR, FTIR, TGA, ^{31}P NMR
33	1-ethyl-3-methylimidazolium	Yes	Clear, gel-like	^{1}H NMR, FTIR, TGA
33	Choline	Yes	Brown, with solid precipitates	FTIR, TGA
34	Alanylglycine	Yes	Clear, viscous	^{1}H NMR, FTIR
**Mixtures**				
35	Glycine, thymine	Yes	Clear, viscous	^{1}H NMR, FTIR
36	Glycine, acetic acid	Yes	Acetic acid evaporates	FTIR
37	Glycine, saccharose	Yes	Dark, Saccharose decomposes	FTIR
38	Glycine, thymine, stearic acid, paraffin, naphthalene	Yes	Dark, semi-solid	FTIR

Table S2. Summary of experiments to test the formation of ionic liquid in various starting conditions.

No.	Experiment	Parameters	Analysis
1	Varying glycine concentration in concentrated sulfuric acid	Gly:H_2SO_4 molar ratio of 1:1, 1:2, 1:3, 0.001:1	Sec. S4, Fig. S14, S15, S16
2	Varying H_2SO_4 concentration in water	wt% of H_2SO_4 in H_2O 0%, 5%, 25%, 50%, 75%, 95%	Sec. S4, Fig S13
3	Microscale formation of [glycine$^+$][HSO_4^-] ionic liquid	300 µg of glycine in 20 µL of 98% w/w H_2SO_4, the rest water	Sec. S4, Fig S16
4	[glycine$^+$][HSO_4^-] formation on basalt	Varying concentration of glycine and H_2SO_4; storage in the open, a desiccated container, and evaporator conditions	Sec. S4, Fig S17, S18

S2. Ionic Liquid Formation Methods

We describe the materials used, detailed experimental setup and procedure. See also Methods Section in the main manuscript.

Materials

This paragraph is repeated from the main manuscript for completeness. We purchased sulfuric acid (95-98%), glycine (Cat. No. G7126), histidine (Cat. No. H8000), thymine (Cat. No. T0376),

adenine (Cat. No. A8626), purine (Cat. No. P55805), guanine (Cat. No. G11950), diaminopurine (Cat. No. 247847), tetrabutylammonium hydroxide (Cat. No. 178780), acetic acid (Cat. No. 695092), oxalic acid (Cat. No. 241172), benzoic acid (Cat. No. 242381), succinic acid (Cat. No. S9512), stearic acid (Cat. No. S4751), palmitic acid (Cat. No. P0500), and dimethyl sulfoxide-d_6 (1034240100) from Sigma-Aldrich. The remaining biogenic amino acids—alanine, arginine, asparagine, aspartic acid, cystine, lysine, methionine, tryptophan, valine, glutamine, glutamic acid, isoleucine, leucine, phenylalanine—were part of a kit of 20 amino acids from SigmaAldrich (Cat. No. LAA21-1KT). Choline-hydroxide (Cat. No. 224210) was purchased from Beantown Chemicals and 1-Ethyl-3-methylimidazolium hydrogen sulfate (H27232) and pyrimidine (157700050) from Thermo Scientific Chemicals. The triethylphosphine oxide (Cat. No. AA3039103) was purchased from Fisher Scientific. Alanylglycine dipeptide (Cat. No. ALA121374-5G) and basalt rock samples Ward's Basalt (Cat. No. 470025-914) are purchased from VWR.

The Thermo-vacuum Evaporation Setup

The custom evaporator (placed inside the fume hood) used for the removal of excess sulfuric acid under low pressure is shown in Figure S1. The setup includes the following main components. (1) A rotary vane vacuum pump prepared to be used with corrosion-resistant PFPE oil (Alcatel 2010SD Mechanical Pump). The pump is capable of a base pressure of 0.01 mbar. (2) The vacuum chamber consists of a borosilicate glass jar (VWR Cat. No. 89055-058), resistant to sulfuric acid, and allows observation of the evaporation process. (3) A hot plate with temperature control (VWR Cat. No. 76447-036). We experimentally determined the evaporation rate of 80%, 90%, and 98% w/w sulfuric acid, the rest water (without any dissolved solute) in the custom evaporator (Figure S1). The temperature was varied from 90-125 °C in 5 °C intervals and three data points were obtained at each temperature and concentration condition. For every data point, 20 µL sulfuric acid was pipetted onto a glass dish and the area over which it spread was measured. The pressure reading and time required for complete evaporation of sulfuric acid was calculated. We used this data to further calculate the minimum time required to evaporate sulfuric acid from a mixture of organic and sulfuric acid.

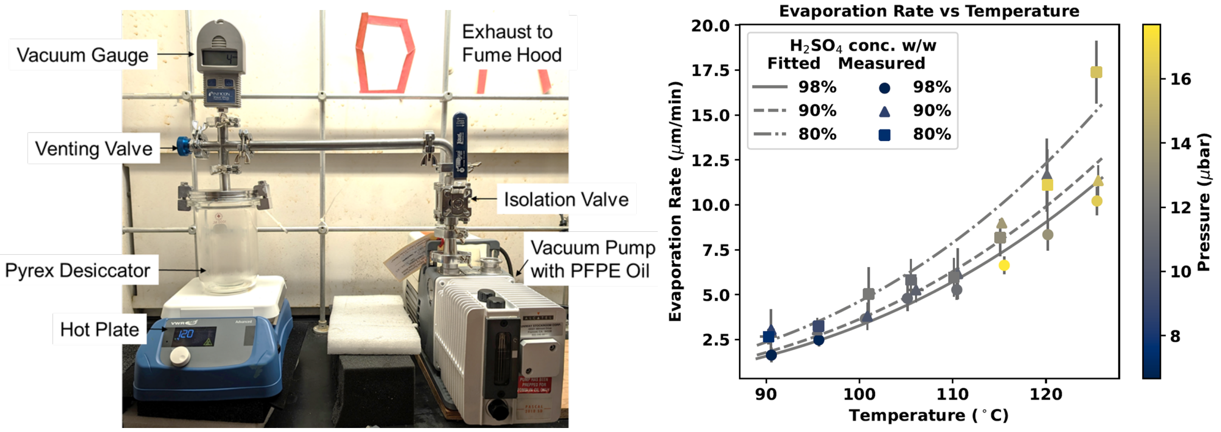

Figure S1. Left panel: Thermo-vacuum evaporation setup with the key components labeled. Right panel: Evaporation rate of sulfuric acid (without any solute) in µm/min (or 10^4×mL/cm^2/min) on the y-axis as a function of temperature in °C on the x-axis. The colorbar on the right indicates the average pressure in µbar inside the chamber for each measurement.

Ionic Liquid Production Process

In this section we summarize the procedure for ionic liquid production. We describe the formation of hydrogen sulfate ionic liquids in equimolar (1:1 molar, also called neat), as well as the non-equimolar variants.

To form equimolar ionic liquids, we mixed approximately equal molar amounts of the organic compound and 98% w/w sulfuric acid (the rest water). We then added about 5% extra pure H_2SO_4 to ensure the complete dissolution of organic molecules in concentrated sulfuric acid. For example, to form an equimolar [glycine$^+$][HSO_4^-] ionic liquid, we mixed 1.4 g of glycine into 1.05 mL of pure 98% w/w H_2SO_4 (the rest water). To ensure complete dissolution of organic compounds in concentrated sulfuric acid we incubated the mixture in a sand bath at 80 °C +/-5 °C for 12-24 hours. This initial incubation time does not affect the ionic liquid formation experiment. Once the organics were completely dissolved, we evaporated the excess sulfuric acid from the mixture inside the custom thermo-vacuum evaporator setup (100 °C +/-5 °C and 0.01 mbar pressure). We chose these temperature-pressure conditions to allow for slow evaporation of excess sulfuric acid. We left the evaporation system running overnight. The exact time did not matter as long as it was long enough to evaporate the excess sulfuric acid. Once the ionic liquid formed, the ionic liquid did not evaporate. To estimate the minimum time to ensure complete evaporation of excess sulfuric acid, we used the evaporation rate data for concentrated sulfuric acid alone (Figure S1) and ran the ionic liquid formation experiment for at least twice the required calculated time. For example, to form an equimolar [glycine$^+$][HSO_4^-] ionic liquid, the extra 50 µL of H_2SO_4, beyond the 1:1 molar ratio, requires 2 hours to evaporate. Therefore, we ran the evaporator overnight (>12 hours) to ensure complete evaporation of excess sulfuric acid. We confirmed that the ionic liquid does not evaporate by leaving a ~10 µL glycine- H_2SO_4 sample in the evaporator (100 °C +/-5 °C and 0.01 mbar pressure) for 24 hours and it does not evaporate.

We follow the same procedure in preparation of all the ionic liquids from all other organic compounds listed in Table S1.

The exact temperature-pressure conditions for preparation of our ionic liquids do not matter as long as the temperature is below the decomposition temperatures of organic compounds (Table 1 in the main manuscript) and the pressure is low enough to ensure evaporation of excess sulfuric acid. See Section S4 for further data on the ionic liquid formation procedures and the estimation of the evaporation rate of concentrated sulfuric acid.

For the non-equimolar ionic liquid mixtures (1:2, 1:3) and the diluted glycine sample (0.001:1) we followed the same procedure as for the 1:1 equimolar ionic liquid formation. We adjusted the required volume of liquid concentrated sulfuric acid accordingly (Table S3). See Section S4 for further data on the ionic liquid formation procedures and the estimation of the evaporation rate of concentrated sulfuric acid.

Table S3. Amounts of glycine and concentrated sulfuric acid (98% w/w H_2SO_4, the rest water) used in ionic liquid formation.

Molar Ratio	Glycine (mg) (conc. in M)	H_2SO_4 (mL) Before Evaporation
1:1	1400 (~18M)	1.05
0.001:1	1.4 (20mM)	1
1:2	1400 (~9M)	2
1:3	1400 (~6M)	3

S3. Ionic Liquid Chemical Analysis Methods and Results

In this section we provide further details on ionic liquid chemical characterization and analysis. We describe the methods and additional supplementary results for ^{1}H NMR, FTIR, TGA and acidity assessment (^{31}P NMR) for a set of selected ionic liquids (Table S1 and Figure S2).

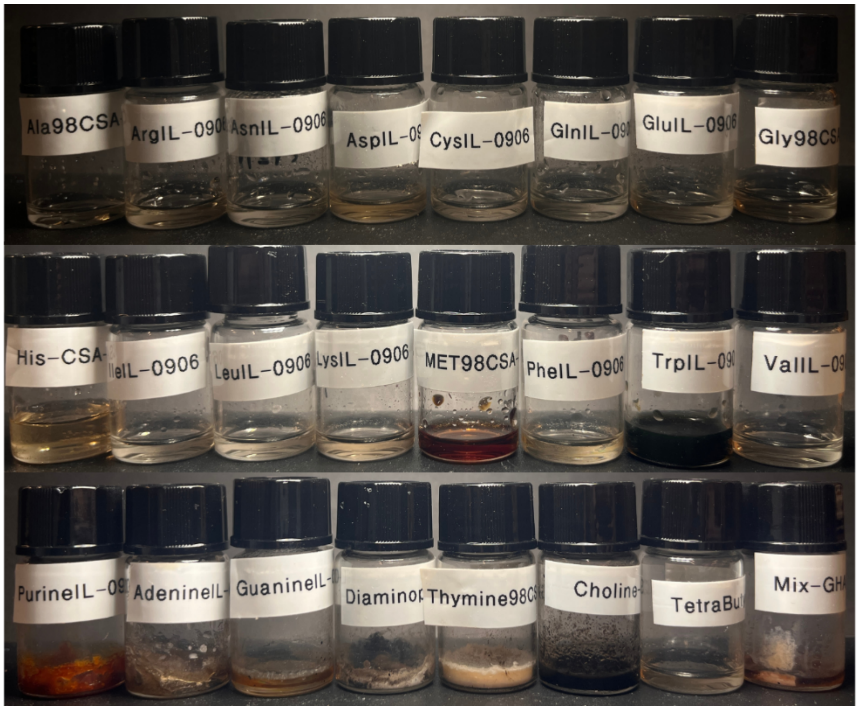

Figure S2. Vials of evaporation products composed of N-containing organics and hydrogen sulfate (HSO_4^-) anions. The starting organic compounds are labeled on each vial. The ionic liquids were generated by evaporating excess sulfuric acid from mixtures of various organic compounds and 98% w/w sulfuric acid (the rest water). Note that even compounds that are unstable in concentrated sulfuric acid, like tryptophan (6), that react to form a highly complex mixture of diverse organics, do form ionic liquids upon evaporation of excess sulfuric acid.

^{1}H Nuclear Magnetic Resonance (NMR) Spectroscopy

This paragraph is repeated from the main manuscript and expanded upon. We use ^{1}H NMR spectra to further support the characterization of the selected ionic liquids. The neat ionic liquids were transferred to NMR tubes (5 mm, borosilicate glass). Approximately 300-400 µL ionic liquid was pipetted into the NMR tubes. A sealed capillary tube containing a deuterated solvent dimethyl sulfoxide-d_6 (DMSO-d_6), as an external lock, was inserted into the NMR tube containing the ionic liquid. We obtained the ^{1}H NMR spectra at 80 °C using a Bruker Avance Neo spectrometer operating at 500.18 MHz (Figure S3 and Figure S4).

We assigned the peaks corresponding to SO-H and N-H for each tested ionic liquid (Table S4). In all the spectra, the SO-H peak is the most downfield shifted. For all NMR-measured ionic liquids (with the exception of tetrabutylammonium and tryptophan ionic liquid samples) we identify two sets of protons in the 6-12 ppm region of the ^{1}H NMR spectrum that are characteristic of cationic and anionic species. For the [glycine$^+$][HSO_4^-] ionic liquid the most downfield-shifted peak, at 10.26 ppm, corresponds to the SO-H proton of [HSO_4^-] anion while the peak at 6.18 ppm corresponds to the amine hydrogens of the protonated glycine molecule. We note that the tetrabutylammonium$^+$ [TBA$^+$] cation does not have a N-H group; thus, no N-H peak is visible.

We included the tryptophan-based ionic liquid to show that even in a complex mixture of many N-containing organic compounds, an ionic liquid will still form upon evaporation of excess sulfuric acid (Figure S2 and Figure S3). Tryptophan is unstable and reacts readily with concentrated sulfuric acid (6). The ionic liquid that forms from tryptophan and sulfuric acid is a product of that reactivity. The resulting ^{1}H NMR therefore is very “crowded” as it contains many different reaction products. The identity of the positively charged organic molecule that forms the ionic liquid is unknown, but it has to be one, or more, product of tryptophan decomposition. The identification of these products is beyond the scope of this work.

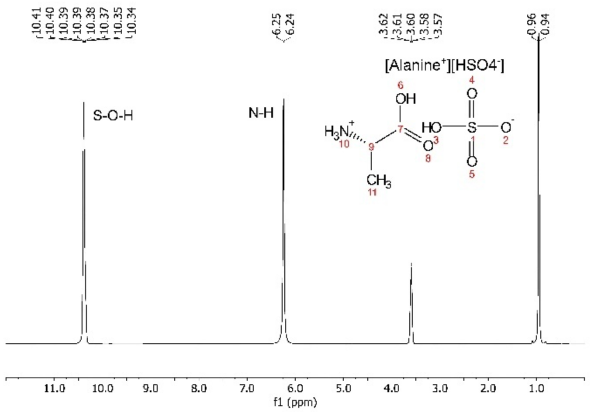
10.41 10.40 10.39 10.39 10.38 10.37 10.35 10.34
6.25 6.24
3.62 3.61 3.60 3.58 3.57
0.96 0.94
[Alanine+][HSO4-]
S-O-H
N-H
f1 (ppm)

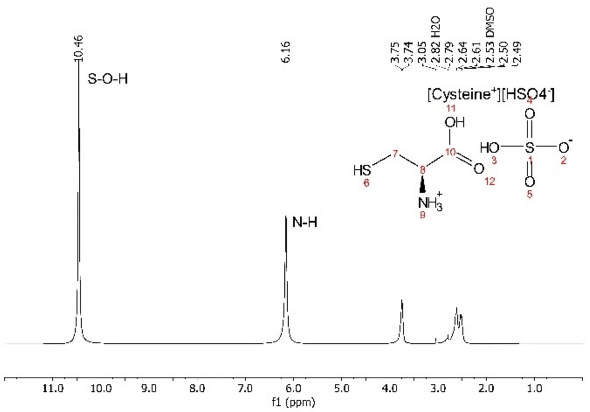
10.46
6.16
3.75 3.74 3.05 2.82 H2O 2.79 2.64 2.61 2.53 DMSO 2.50 2.49
[Cysteine+][HSO4-]
S-O-H
N-H
f1 (ppm)

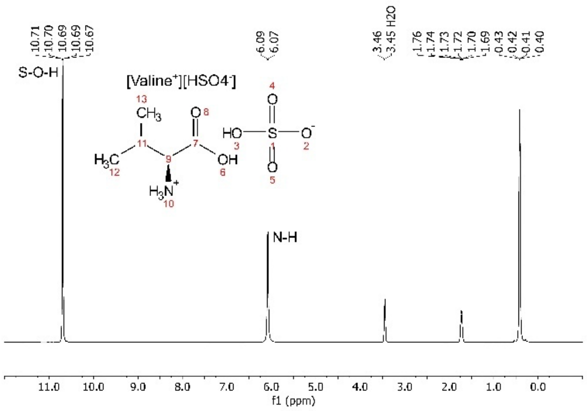
10.71 10.70 10.69 10.69 10.67
6.09 6.07
3.46 3.45 H2O
1.76 1.74 1.73 1.72 1.70 1.69 0.43 0.42 0.41 0.40
[Valine+][HSO4-]
S-O-H
N-H
f1 (ppm)

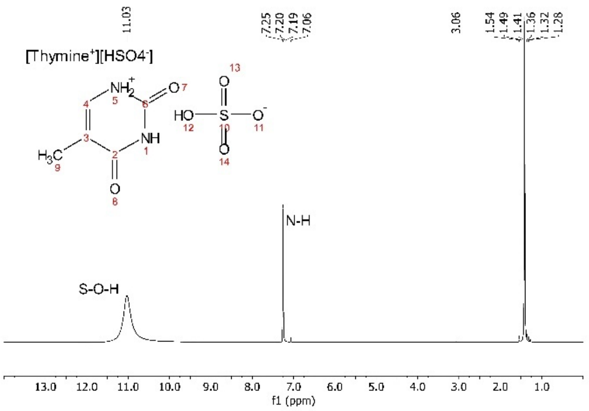
11.03
7.25 7.20 7.19 7.06
3.06
1.54 1.49 1.41 1.36 1.32 1.28
[Thymine+][HSO4-]
S-O-H
N-H
f1 (ppm)

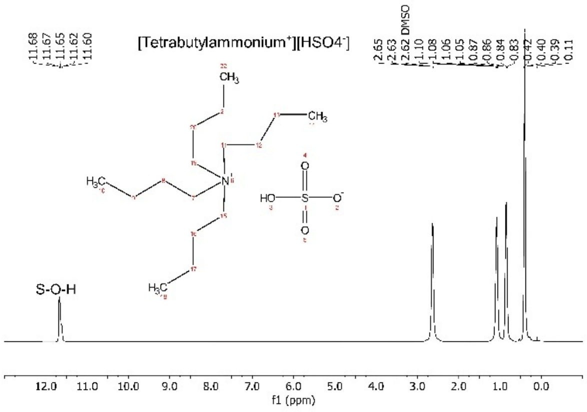
11.68 11.67 11.65 11.62 11.60
2.65 2.63 2.62 DMSO 1.10 1.08 1.06 1.05 0.87 0.86 0.84 0.83 0.42 0.40 0.39 0.11
[Tetrabutylammonium+][HSO4-]
S-O-H
f1 (ppm)

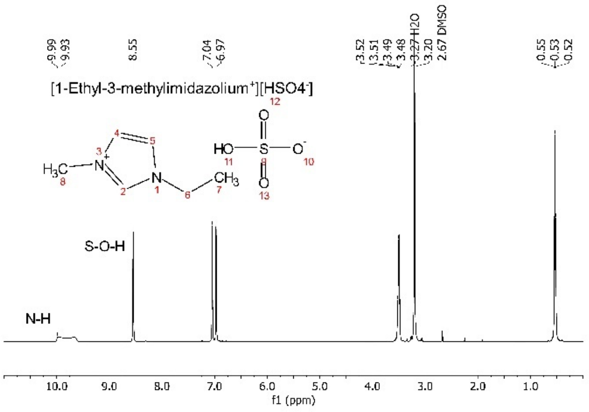
9.99 9.93
8.55
7.04 6.97
3.52 3.51 3.49 3.48 3.27 H2O 3.20 2.67 DMSO
0.55 0.53 0.52
[1-Ethyl-3-methylimidazolium+][HSO4-]
S-O-H
N-H
f1 (ppm)

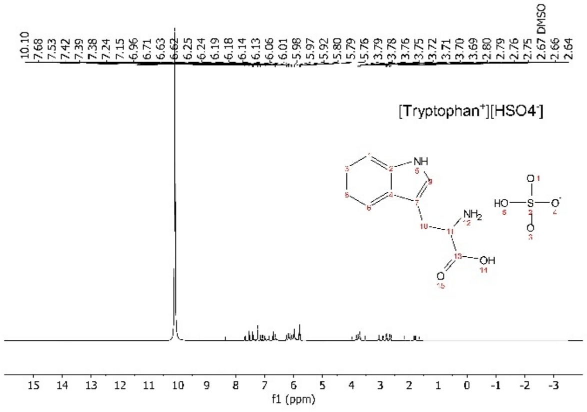
10.10 7.68 7.53 7.42 7.39 7.38 7.24 7.15 6.96 6.71 6.63 6.62 6.25 6.24 6.19 6.18 6.14 6.13 6.06 6.01 5.98 5.97 5.92 5.80 5.79 5.76 3.79 3.78 3.76 3.75 3.72 3.71 3.70 3.69 2.80 2.79 2.76 2.75 2.67 DMSO 2.66 2.64
[Tryptophan+][HSO4-]
f1 (ppm)

Figure S3. ^{1}H NMR spectra of selected ionic liquids formed from various N-containing organics dissolved in concentrated sulfuric acid (98% H_2SO_4 w/w, the rest water) in equimolar (i.e., 1:1) concentration after the evaporation procedure. The spectra were recorded at 80 °C and 500.18 MHz with DMSO-d_6 as an external lock solvent. Note that even the unstable and reactive organics, like tryptophan, form ionic liquids with concentrated sulfuric acid.

Table S4. ^{1}H NMR peaks of selected ionic liquids with signals assigned to N-H and S-O-H protons.

Sample	δN-H(ppm)	δSO-H(ppm)
[glycine$^+$][HSO_4^-]	6.18	10.26
[glycine$^+$][HSO_4^-][H_2SO_4]	6.03	10.35
[glycine$^+$][HSO_4^-][H_2SO_4]$_2$	5.95	10.37
[alanine$^+$][HSO_4^-]	6.25	10.39
[cysteine$^+$][HSO_4^-]	6.16	10.46
[valine$^+$][HSO_4^-]	6.09	10.69
[tryptophan products$^+$][HSO_4^-]	-	10.10
[thymine$^+$][HSO_4^-]	7.20	11.03
[tetrabutylammonium$^+$][HSO_4^-]	-	11.65
[1-ethyl-3-methylimidazolium$^+$][HSO_4^-]	~10.00	~10.00
[glycine$^+$][HSO_4^-][thymine$^+$][HSO_4^-]	6.17, 7.15	10.22
[ala-gly$^+$][HSO_4^-]	6.30	10.51

Apart from amino acids, we also tested simple peptides like the alanylglycine (Ala-Gly) dipeptide for their ionic liquid formation capability (Figure S4). We note that Ala-Gly is stable in 98% sulfuric acid (the rest water) at room temperature for many months (7). Ala-Gly forms ionic liquid after heating to 80 °C for 24h followed by evaporation of excess sulfuric acid (see Section S2 for ionic liquid formation method and Section S6).

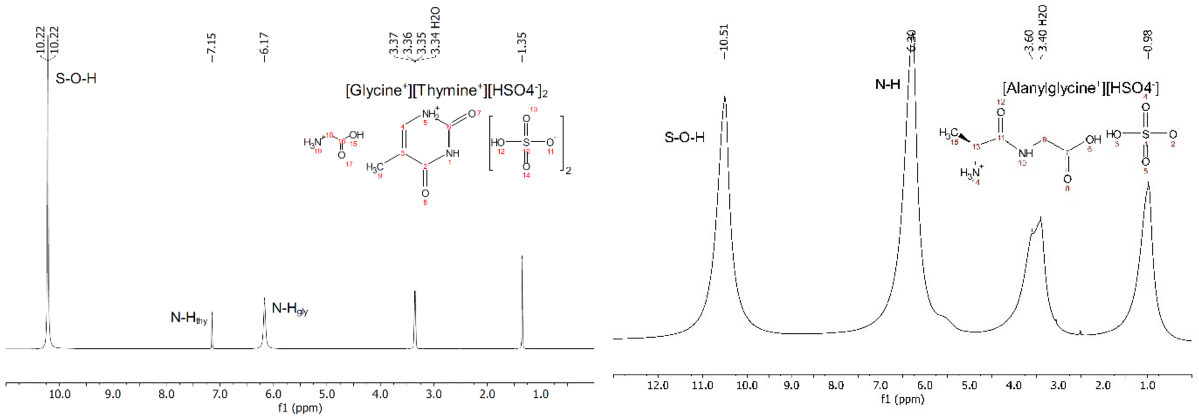

Figure S4. ^{1}H NMR spectra of ionic liquid formed from a mixture of glycine and thymine in concentrated sulfuric acid (98% H_2SO_4 by weight, the rest water) in molar ratio of 1:1:2 of glycine:thymine:H_2SO_4 (left panel) and alanylglycine dipeptide ionic liquid (right panel). The spectra were recorded at 80 °C and 500.18 MHz with DMSO-d_6 as an external lock solvent.

Fourier-Transform Infrared (FTIR) Spectroscopy

We used FTIR spectroscopy to characterize selected ionic liquids (Figure S5) following the example of [glycine$^+$][HSO_4^-] ionic liquid described in the main text (Figure 3). We compared the IR spectra of [glycine$^+$][HSO_4^-] ionic liquid with the IR spectra of pure glycine and pure 98% w/w H_2SO_4 (the rest water) respectively. The carboxylate ion [COO^-] stretching in pure glycine appears between 1400 and 1600 cm^{-1}. In the ionic liquid, these bands shift to a higher wavenumber corresponding to [COOH] group vibration. The pure H_2SO_4 has a prominent peak at 1345 cm^{-1}, corresponding to S-OH bending vibrations. These vibrations are not prominent in the ionic liquid due to the strong ionic bonds. The [glycine$^+$][HSO_4^-] ionic liquid IR spectra are consistent with the IR spectra of hydrogen sulfate ionic liquids reported in the literature (8). Other tested ionic liquids exhibit similar features characteristic for hydrogen sulfate ionic liquids (Table S5).

This paragraph is repeated from the main manuscript and expanded upon. We obtained the infrared spectra of ionic liquids and pure forms of the constituents using a Bruker Alpha II FTIR spectrometer with a Diamond Crystal ATR (Attenuated Total internal Reflectance) accessory. We acquired 64 scans for each sample with 2 cm^{-1} resolution. For each measurement of the ionic liquids, about ~5 µL liquid was transferred to the ATR crystal using a pipette. The liquid was exposed to air for 5-10 minutes while the spectra were obtained. Since the ionic liquids are highly hygroscopic (see Figure S11), this exposure to air in addition to variations in humidity during storage could lead to some absorption of water in the ionic liquids. This is a possible explanation for the shoulder around 3500 cm^{-1} seen in a few of the IR spectra.

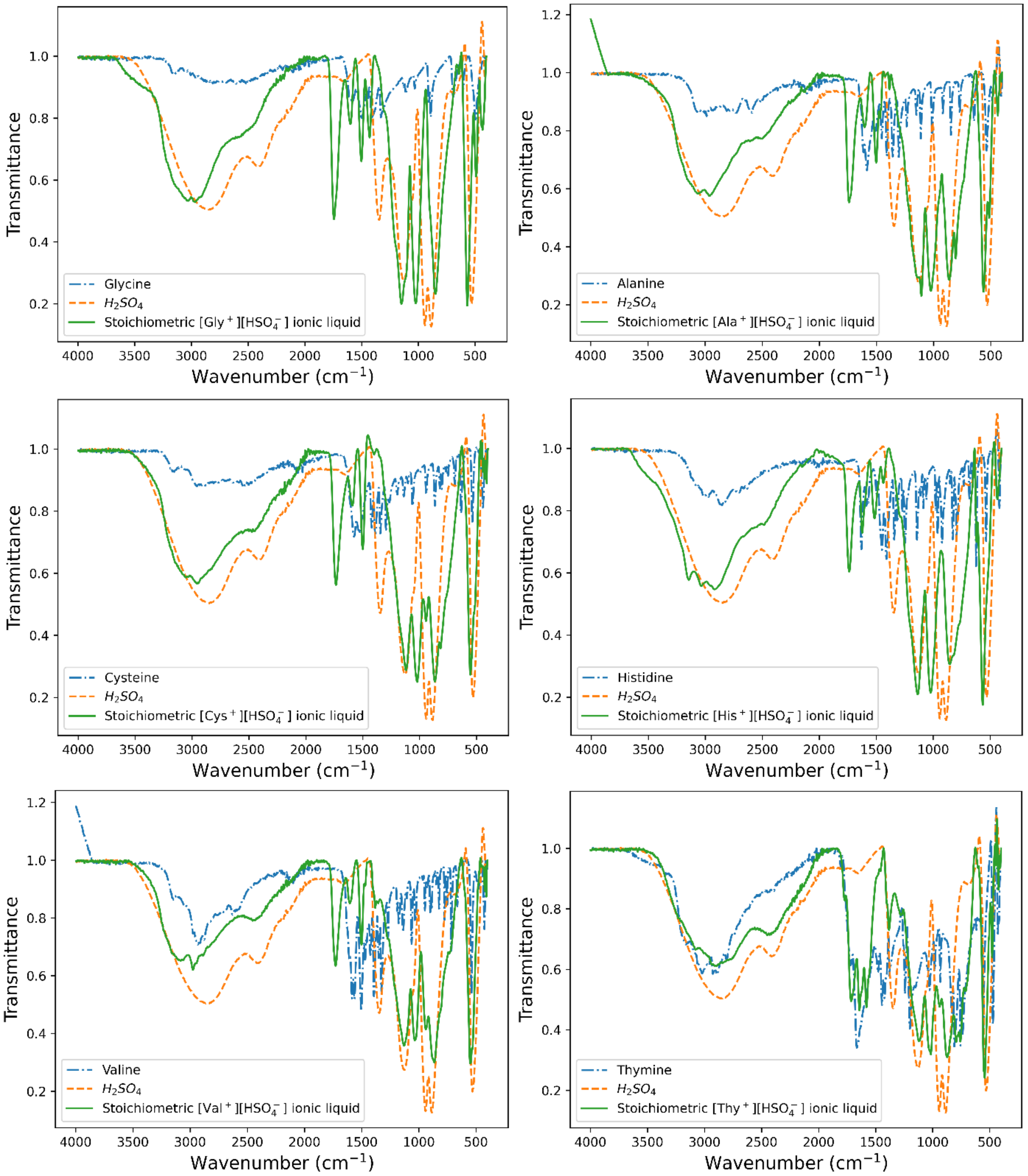

Transmittance
Wavenumber (cm^{-1})
Glycine
H_2SO_4
Stoichiometric [Gly$^+$][HSO_4^-] ionic liquid
Alanine
H_2SO_4
Stoichiometric [Ala$^+$][HSO_4^-] ionic liquid
Cysteine
H_2SO_4
Stoichiometric [Cys$^+$][HSO_4^-] ionic liquid
Histidine
H_2SO_4
Stoichiometric [His$^+$][HSO_4^-] ionic liquid
Valine
H_2SO_4
Stoichiometric [Val$^+$][HSO_4^-] ionic liquid
Thymine
H_2SO_4
Stoichiometric [Thy$^+$][HSO_4^-] ionic liquid

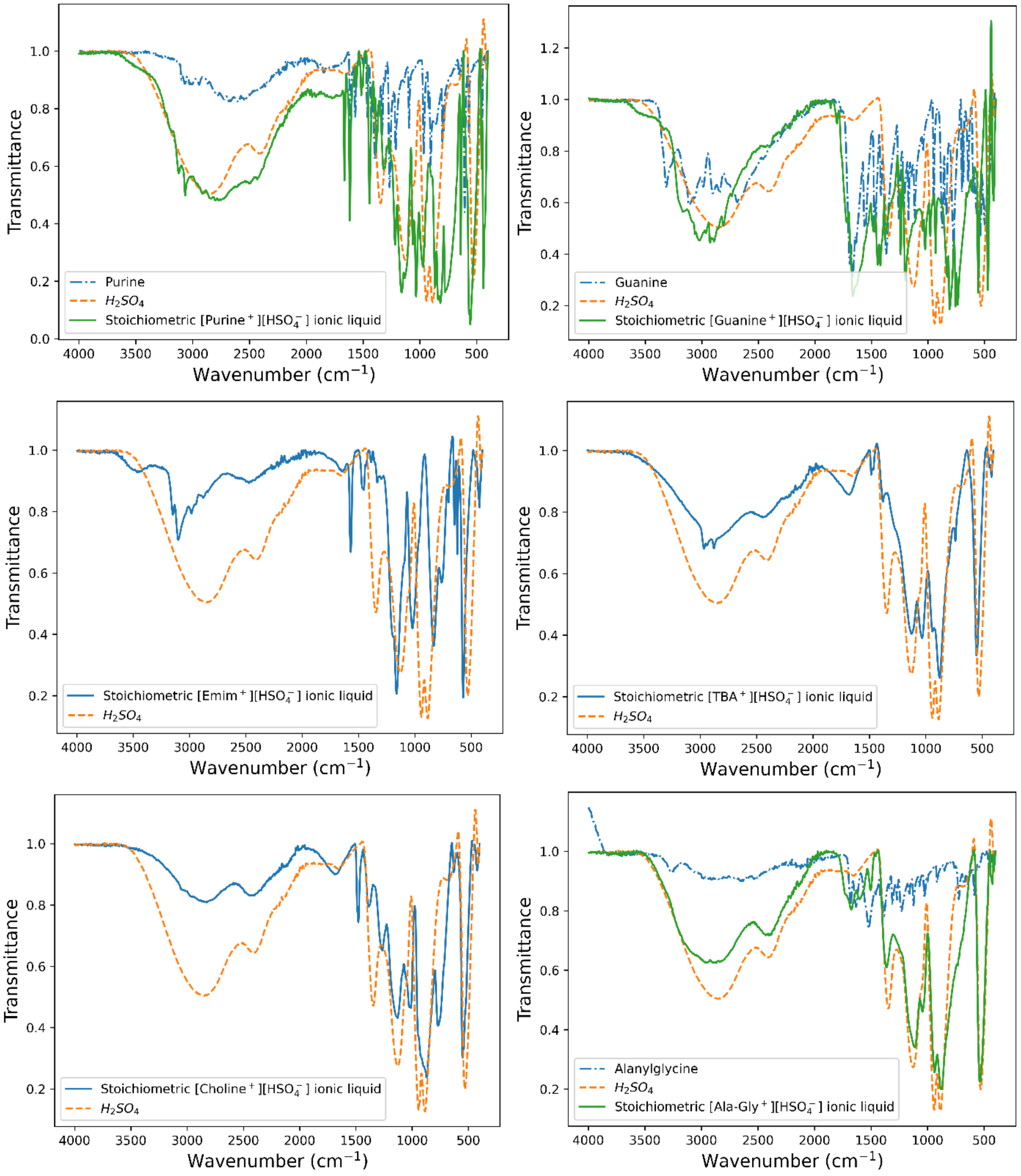

Figure S5. The FTIR spectra of selected ionic liquids formed from various N-containing organics dissolved in concentrated sulfuric acid (98% H_2SO_4 by weight, the rest water), in equimolar (i.e., 1:1) concentration, after the evaporation procedure. The y-axis is transmittance and the x-axis is wavenumber in cm^{-1}. (Emim is 1-ethyl-3-butylimidazolium; TBA is tetrabutylammonium).

Table S5. FTIR spectra peak assignments for the generated ionic liquids and H_2SO_4, compared with the peaks from [2].

Sample	S-O		S=O			S-OH
	$[HSO_4^-]$	H_2SO_4	$[HSO_4^-]$	$[HSO_4^-]$	H_2SO_4	H_2SO_4
H_2SO_4	884.03	940.36	1047.10	-	1123.30	1345.04
[glycine$^+$][HSO_4^-]	852.01	-	1020.30	1139.57	-	-
[glycine$^+$][HSO_4^-][H_2SO_4]	853.46	-	1015.37	-	1103.47	-
[glycine$^+$][HSO_4^-][H_2SO_4]$_2$	877.11	939.56	1027.32	-	1101.25	-
[histidine$^+$][HSO_4^-]	851.30	-	1022.78	1135.35	-	-
[alanine$^+$][HSO_4^-]	861.19	-	1025.21	-	1107.00	-
[cysteine$^+$][HSO_4^-]	865.19	940.97	1020.96	1120.09	-	-
[valine$^+$][HSO_4^-]	864.89	-	1034.35	1130.21	-	-
[thymine$^+$][HSO_4^-]	853.30	-	1011.00		1106.00	
[purine$^+$][HSO_4^-]	864.95	-	1034.02	1160.79	-	1320.67
[guanine$^+$][HSO_4^-]	809.41	933.44	1026.01	1200	-	-
[1-ethyl-3-methylimidazolium$^+$][HSO_4^-]	831.74	-	1023.56	1163.25	-	-
[tetrabutylammonium$^+$][HSO_4^-]	875.29	-	1032.07	1127.38	-	-
[choline$^+$][HSO_4^-]	871.27	-	1011.78	1128.69	-	1383.10
[alanylglycine$^+$][HSO_4^-]	875.78	936.86	1045.31	-	1111.18	1363.67
[glycine$^+$][HSO_4^-][thymine$^+$][HSO_4^-]	857.59	941.01	1021.92		1106.10	-
Organic mixture 1	832.67	-	1022.32	1145.59	-	-
H_2SO_4 (8)	885.60	942.70	1047.10	-	1125.50	1347.50
[Hmpyr$^+$][(HSO_4)(H_2SO_4)0.5] (8)	844.20	-	1020.50	1150.80	-	-

The comparison of the IR spectra of the [glycine$^+$][HSO_4^-] ionic liquid with the spectra of the liquid that arises from evaporating glycine (280 mg) and acetic acid (300 μL) dissolved in 450 μL 98% w/w H_2SO_4 (the rest water) show that the two spectra match (Figure S6), confirming that acetic acid evaporates from the mixture and does not inhibit the formation of [glycine$^+$][HSO_4^-] ionic liquid. We additionally tested a mixture of glycine and thymine in concentrated sulfuric acid (in molar ratio 1:1:2 of gly:thy:H_2SO_4) and found that such a mixture also formed ionic liquid (Figure S7). In contrast to the pure [thymine$^+$][HSO_4^-] salt that is solid at room temperature (but liquid at 100 °C) the mixture of glycine and thymine forms a room temperature ionic liquid.

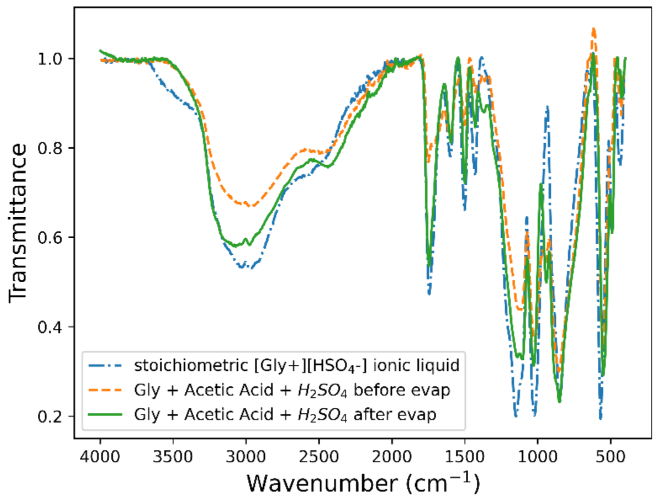

Figure S6. FTIR spectrum of [glycine$^+$][HSO$_4^-$] ionic liquid (blue) compared with the ionic liquid generated from a glycine, acetic acid and H_2SO_4 mixture (orange). The y-axis is transmittance and the x-axis is wavenumber in cm^{-1}. The two spectra match, showing that acetic acid evaporates from the mixture and does not inhibit the formation of [glycine$^+$][HSO$_4^-$] ionic liquid.

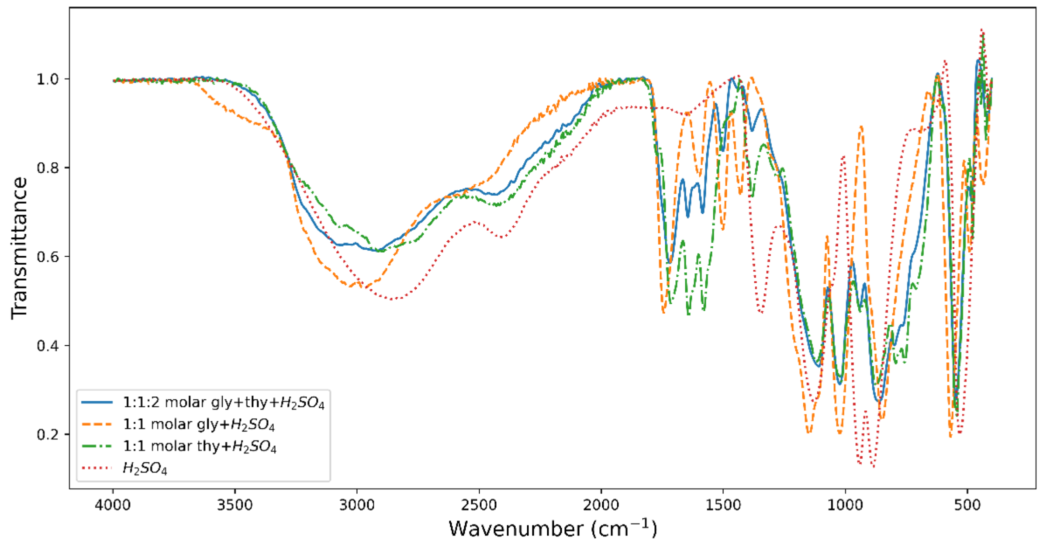

Figure S7. FTIR spectrum of a mixture of glycine and thymine-based ionic liquids with concentrated sulfuric acid. The y-axis is transmittance and the x-axis is wavenumber in cm^{-1}. The FTIR spectra of neat ionic liquids [glycine$^+$][HSO$_4^-$] (orange spectra), [thymine$^+$][HSO$_4^-$] (green spectra) compared to [glycine$^+$][thymine$^+$][HSO$_4^-$] (blue spectra), a mixture of the two organic components with concentrated sulfuric acid (98% H_2SO_4 w/w, the rest water) in molar ratio of 1:1:2 of glycine:thymine:H_2SO_4. In contrast to [thymine$^+$][HSO$_4^-$] the mixture of glycine and thymine forms a room temperature ionic liquid.

Other tested mixtures include a mixture of glycine (114 mg) and sugar (112 mg) in 400 µL concentrated sulfuric acid (Figure S8). Another mixture contained N-containing organics, carboxylic acids, and hydrocarbons. The mixture was prepared by mixing glycine (10 mg), thymine (10 mg), stearic acid (50 mg), paraffin (wax) (5 mg), and naphthalene (5 mg), in 1 mL concentrated sulfuric acid (98% w/w H_2SO_4 the rest water) (Figure S9). The sugar, wax, and

naphthalene were household items. The spectra before evaporation has dominant H_2SO_4 features and matches closely with the pure H_2SO_4 spectra. The spectra after evaporation shows a rightward shift in the S-O vibration region corresponding to the HSO_4^- anion, consistent with other ionic liquids (Table S5).

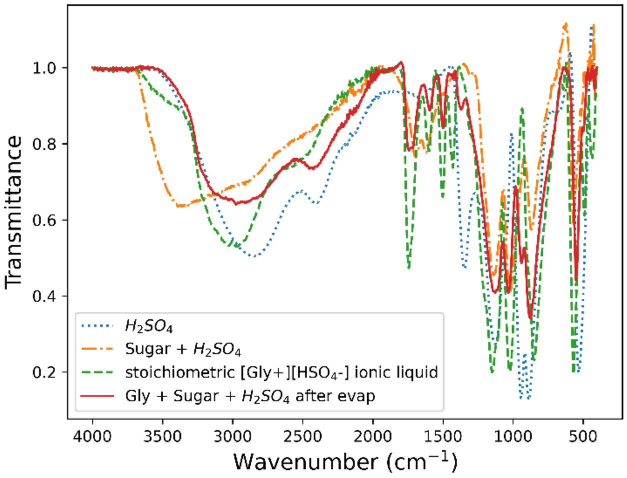

Figure S8. FTIR spectrum of [glycine$^+$][HSO_4^-] ionic liquid (green) compared with the ionic liquid generated from a glycine, sugar and H_2SO_4 mixture (red). The y-axis is transmittance and the x-axis is wavenumber in cm^{-1}. The bands corresponding to [glycine$^+$] (1400-1500 cm^{-1} region) and [HSO_4^-] ions (850-1100 cm^{-1} region) match, showing that sugar does not inhibit the formation of [glycine$^+$][HSO_4^-] ionic liquid but reacts with the excess sulfuric acid.

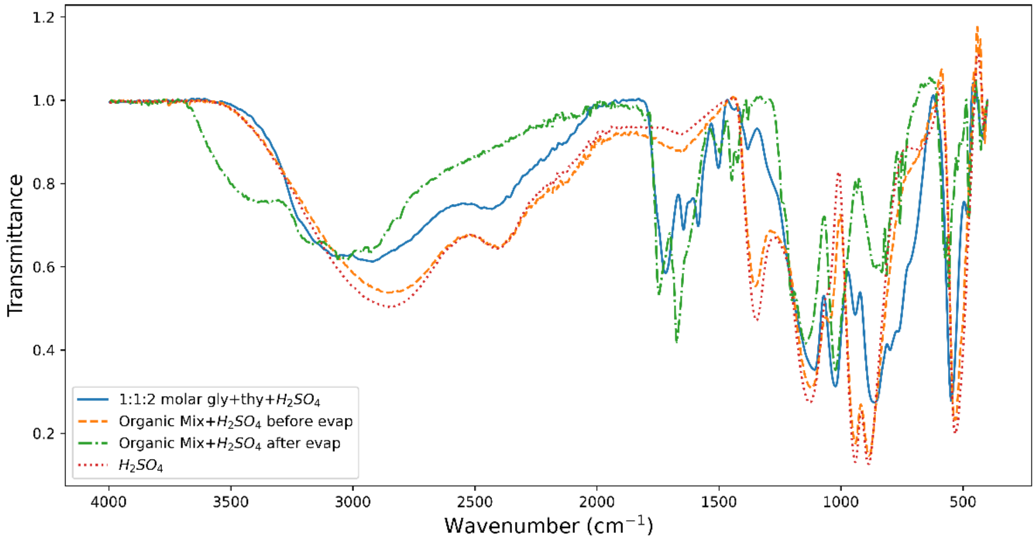

Figure S9. FTIR spectrum of an organic mixture (glycine, thymine, stearic acid, paraffin, naphthalene) in 98% w/w sulfuric acid, the rest water. The y-axis is transmittance and the x-axis is wavenumber in cm^{-1}. The IR spectra of the mixture before and after evaporation of the excess sulfuric acid differs significantly. The evaporation of the complex mixture of organics also results in the formation of ionic liquid.

Thermogravimetric Analysis (TGA)

We used the TGA method to determine the onset temperature of thermal decomposition of selected ionic liquids. We found that the tested ionic liquids have various decomposition

temperatures depending on component molecules and mixtures, but in all cases the decomposition temperatures are higher than about 180–190 ℃ (Figure S10 and Table 1 in the main text).

This paragraph is repeated from the main manuscript. About 1-2 mg of neat ionic liquid was loaded onto a platinum pan. The samples were gradually heated from 30 °C to 600 °C with a rate of 10 °C /min under the flow of nitrogen. The weight change was measured using TA Instruments Discovery TGA 5500.

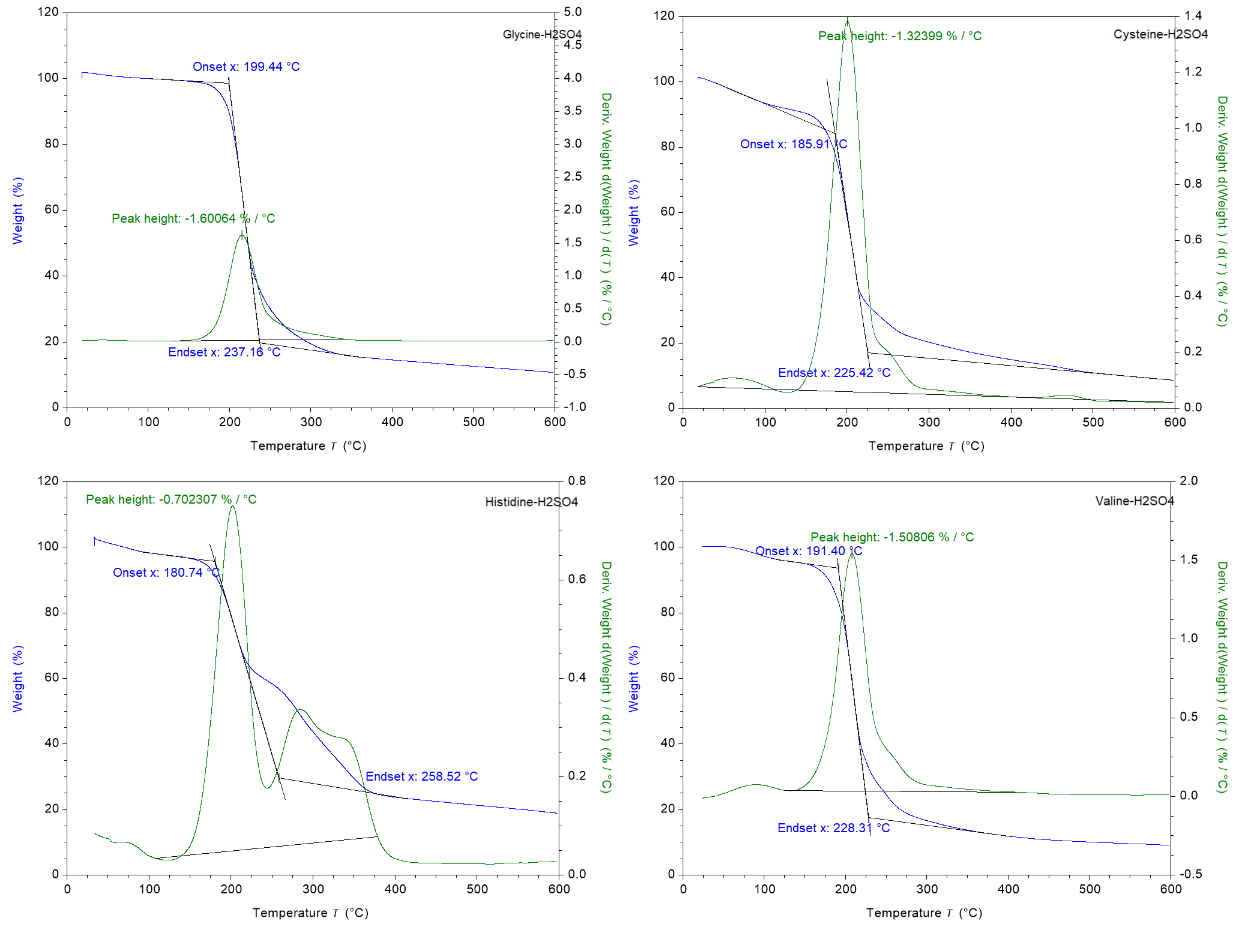

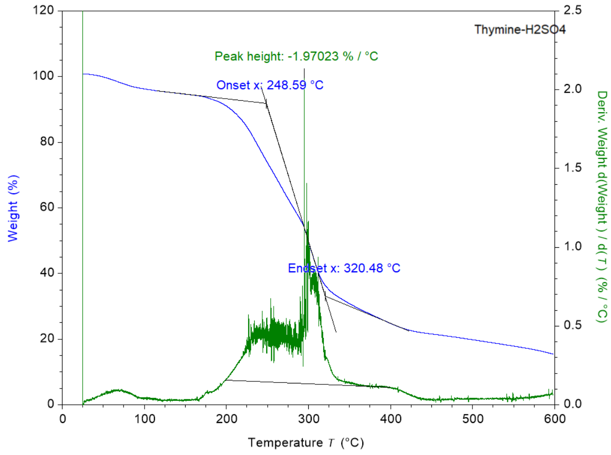
Thymine-H2SO4
Peak height: -1.97023 % / °C
Onset x: 248.59 °C
Endset x: 320.48 °C
Weight (%)
Deriv. Weight d(Weight) / d(T) (% / °C)
Temperature T (°C)

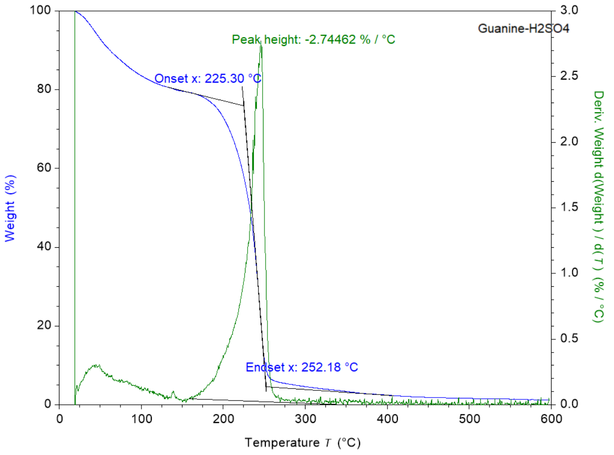
Guanine-H2SO4
Peak height: -2.74462 % / °C
Onset x: 225.30 °C
Endset x: 252.18 °C
Weight (%)
Deriv. Weight d(Weight) / d(T) (% / °C)
Temperature T (°C)

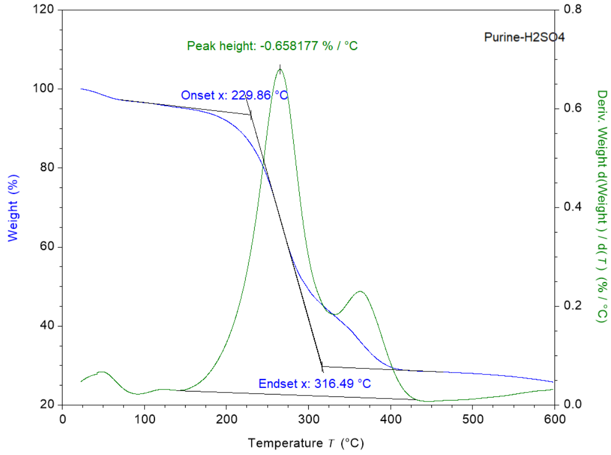
Purine-H2SO4
Peak height: -0.658177 % / °C
Onset x: 229.86 °C
Endset x: 316.49 °C
Weight (%)
Deriv. Weight d(Weight) / d(T) (% / °C)
Temperature T (°C)

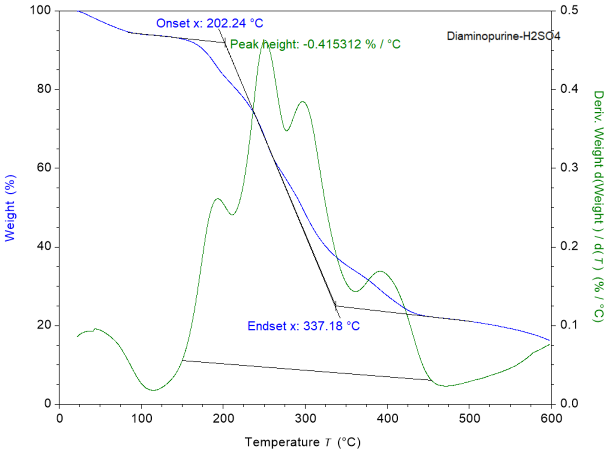
Diaminopurine-H2SO4
Onset x: 202.24 °C
Peak height: -0.415312 % / °C
Endset x: 337.18 °C
Weight (%)
Deriv. Weight d(Weight) / d(T) (% / °C)
Temperature T (°C)

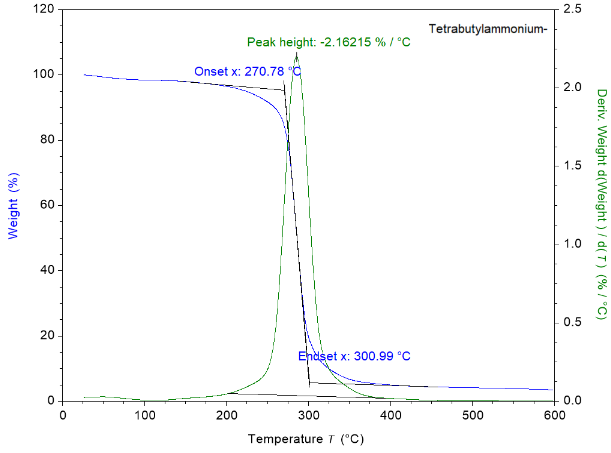
Tetrabutylammonium-
Peak height: -2.16215 % / °C
Onset x: 270.78 °C
Endset x: 300.99 °C
Weight (%)
Deriv. Weight d(Weight) / d(T) (% / °C)
Temperature T (°C)

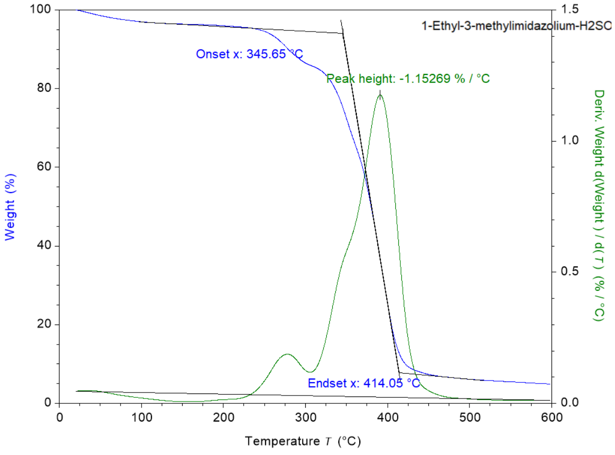
1-Ethyl-3-methylimidazolium-H2SO
Onset x: 345.65 °C
Peak height: -1.15269 % / °C
Endset x: 414.05 °C
Weight (%)
Deriv. Weight d(Weight) / d(T) (% / °C)
Temperature T (°C)

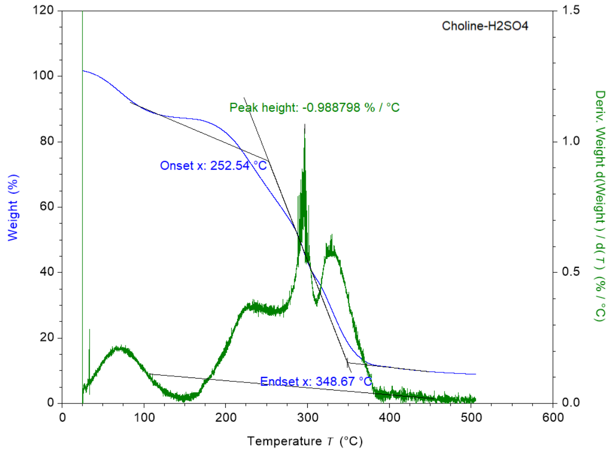

Figure S10. Thermogram curves of selected equimolar ionic liquids containing various N-containing organic molecules. The x-axis is temperature and the left-side y-axis is sample weight % remaining, while the right-side y-axis is first derivative of the weight % with temperature. The temperatures corresponding to the onset, peak, and endset of decomposition are plotted on the curves. The tested ionic liquids have various decomposition temperatures depending on the component organic molecule, but in all cases the decomposition temperature is higher than about 180–190 °C (see also Table 1 in the main text).

^{31}P NMR Acceptor Number Determination

The hydrogen sulfate ionic liquids are acidic in nature (8). We use the Gutmann Beckett Acceptor Number (AN) ^{31}P NMR method to determine the Lewis acidity of selected ionic liquids (9, 10). We show that, as expected, all tested hydrogen sulfate ionic liquids are quite acidic. The [tetrabutylammonium$^+$][HSO_4^-] ionic liquid is the least acidic of the tested substances (AN = 98) (Figure S11 and Table S6). In all cases, however, the acidity of ionic liquids is lower than the acidity of sulfuric acid (AN = 122).

This paragraph is repeated from the main manuscript and expanded upon. We used triethylphosphine oxide (TEPO) as the probe for ^{31}P NMR. Approximately 50 mg of TEPO was added to ~1 ml of the ionic liquid and incubated at 80 °C to allow mixing. The samples were transferred into 5 mm NMR tubes, each with a capillary tube insert containing DMSO-d_6 as external lock. ^{31}P NMR spectra was obtained at 80 °C using a Bruker Avance Neo spectrometer operating at 500.18 MHz. We measured the spectra for H_2O and H_2SO_4 to establish the range of acidity within which the ionic liquid acidity must lie (Figure S11). Table S6 lists the ^{31}P NMR shift of TEPO (δET$_3$PO) and the corresponding acceptor number. The acceptor number is calculated by using the formula: AN = 2.218 × (δET$_3$PO - 41)

We also performed additional AN estimates to confirm the complete evaporation of excess sulfuric acid and the formation of equimolar ionic liquid. We prepared an equimolar ionic liquid by directly adding equal molar amounts of glycine and H_2SO_4 (1400 mg of glycine and 1.037 ml of 98% w/w H_2SO_4 (the rest water), without extra H_2SO_4 and without the evaporation step. This equimolar mixture has an AN of 112.8, a value very similar to the AN value of [glycine$^+$][HSO_4^-] ionic liquid obtained by the evaporation of excess sulfuric acid (AN = 113.1). The obtained AN values further confirm efficient removal of the excess sulfuric acid and the formation of the equimolar

[glycine$^+$][HSO_4^-] ionic liquid. The AN values also confirm the high acidity of the [glycine$^+$][HSO_4^-] ionic liquid.

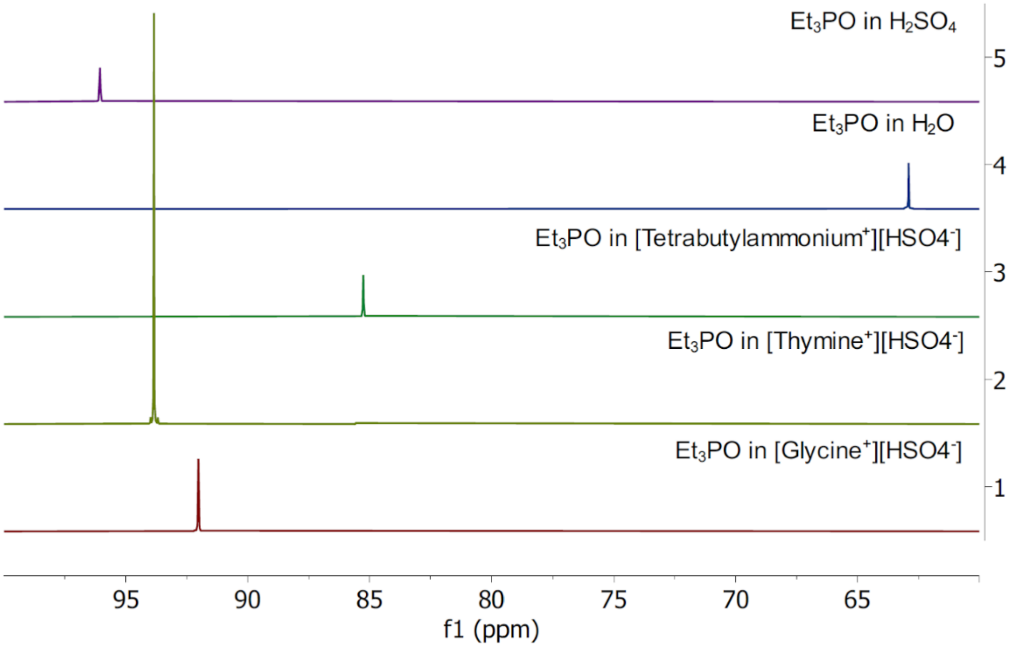

Figure S11. ^{31}P NMR spectra of triethylphosphine oxide (TEPO) dissolved in selected ionic liquids at 80 °C and 500.18 MHz, with DMSO-d$_6$ external lock. The [tetrabutylammonium$^+$][HSO_4^-] ionic liquid is the least acidic of the tested ionic liquids.

Table S6. The ^{31}P NMR shift of TEPO and corresponding acceptor number of select ionic liquids, 98% w/w concentrated H_2SO_4 (the rest water), and pure H_2O.

Sample	δET$_3$PO (ppm)	Acceptor Number
[glycine$^+$][HSO_4^-]	92	113.1
[thymine$^+$][HSO_4^-]	93.8	117.1
[tetrabutylammonium$^+$][HSO_4^-]	85.2	98.0
H_2O	62.9	48.6
98% w/w H_2SO_4 in H_2O	96	122.0

Hygroscopicity

Ionic liquids are very hygroscopic. The [glycine$^+$][HSO_4^-] ionic liquid accumulates a significant amount of water from the surrounding air when left in an open container. A ~1.4 gram of ionic liquid gained 0.34 gram in mass in 14 days (Figure S12). For example, to test how much water the [glycine$^+$][HSO_4^-] ionic liquid accumulates, we measured the initial weight of the sample right after removing it from the glass vial and placed the sample in an open vial (open to ambient lab conditions). The weight of the sample was measured in regular intervals over 14 days. Due to hygroscopicity of ionic liquids, we store them in airtight glass vials.

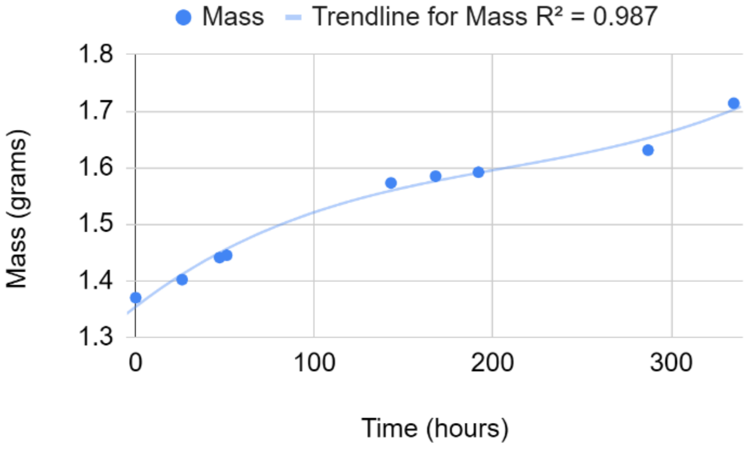

Figure S12. The increase in the mass of the [glycine+][HSO_4^-] ionic liquid due to accumulation of water from the surrounding air. The y-axis is the total mass of the ionic liquid and the x-axis is the time the vial containing an ionic liquid has been left open (in hours). The mass increases monotonically. The non-linear behavior can be attributed to the variation in daily humidity which could cause differences in the rate of accumulation of water over time.

S4. Discussion on Ionic Liquid Formation

Formation of Ionic Liquid with Varying Concentration of Sulfuric Acid in Water

We show that the starting concentration of sulfuric acid does not affect the formation of ionic liquids. We mixed a fixed quantity of glycine in varying concentrations of sulfuric acid in water by weight (0%, 5%, 25%, 50%, 75%, 95%) (Table S7). We evaporated the samples in our custom-built vacuum chamber at 100 °C (+/- 5 °C) and 0.01 mbar pressure for 12 hours, as described in Section S2. We measured the FTIR spectra of the sample before and after evaporation (Figure S11). In the diluted samples, the O-H peak corresponding to H_2O is clearly visible. The post-evaporation spectra of all the samples with non-zero concentration of H_2SO_4 have no H2O OH peak and are consistent with the equimolar ionic liquid [glycine$^+$][HSO_4^-]. After evaporation, the sample with 0% H_2SO_4 leaves a white dry powder of glycine.

Table S7. Quantities used in the experiment to prepare mixtures of glycine in varying H_2SO_4 concentration by weight in H_2O.

Sample No.	H_2SO_4 Conc. in H_2O w/w%	Glycine (mg)	H_2SO_4 (μL)	H_2O (μL)
1	0	103	0	1000
2	5	98	80	2830
3	25	100	80	450
4	50	105	80	150
5	75	99	80	50
6	95	96	80	0

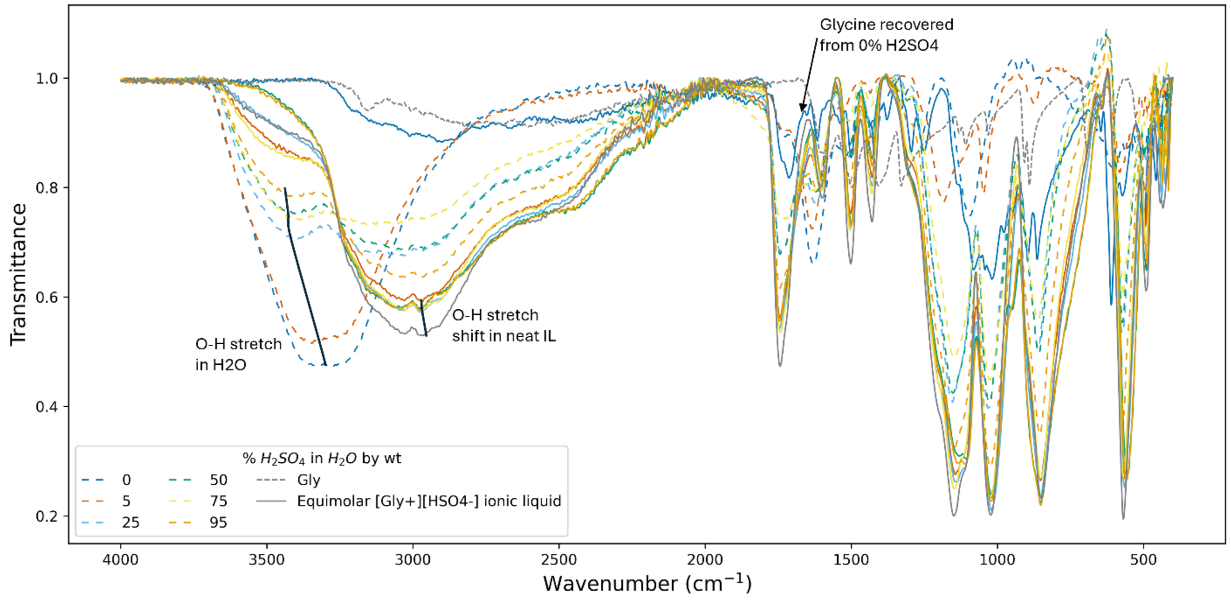

Figure S13. FTIR spectra of ionic liquids formed from mixtures of glycine dissolved in various concentrations of H_2SO_4 in water. The y-axis is transmittance and the x-axis is wavenumber in cm^{-1}. The dashed lines are mixtures before evaporation and the same color solid lines are after evaporation. The FTIR spectra of the evaporated samples with non-zero concentration of H_2SO_4 are consistent with the equimolar ionic liquid [glycine$^+$][HSO_4^-], showing the formation of ionic liquids no matter the starting concentration of sulfuric acid.

Formation of Ionic Liquid with Varying Concentration of Glycine in Sulfuric Acid

Ionic liquids can exist at various molar ratios of cationic and anionic components. Compared to equimolar (1:1) ionic liquids, non-equimolar ionic liquids form by combination of neat ionic liquids with a molar excess of the cationic or anionic species (11). In our exoplanet scenario the abundance of anionic species, i.e. sulfuric acid, will dominate over the available amounts of cationic, organic molecules. Once the excess concentrated sulfuric acid evaporates, the ionic liquid is produced in the equimolar ratio of the cationic and ionic components. Nonetheless, we investigate non-equimolar ratios of the [glycine$^+$][HSO_4^-] (1:2 and 1:3 molar ratios) to further characterize the properties of ionic liquids. We mixed glycine in three molar ratios (1:1, 1:2, 1:3) in concentrated sulfuric acid without evaporation (Section S2 and Table S6).

The spectral characterization of equimolar and non-equimolar ionic liquids has two points of relevance. First, the non-equimolar (1:2 and 1:3) ionic liquids still contain the anionic and cationic components but due to the excess moles of HSO_4^- ions or H_2SO_4 species their FTIR and ^{1}H NMR spectra are different than the equimolar sample. This difference implies that their physicochemical properties are different as well (Figures S14, S15, S16).

Second, the evaporated diluted solution of glycine (20 mM, 0.001:1 molar ratio) dissolved in 98% w/w sulfuric acid (the rest water) reaches the equimolar ratio, thus forming the [glycine$^+$][HSO_4^-] ionic liquid (Figure S16). We show that when a non-equimolar ionic liquid is subjected to temperature and pressure conditions that allow evaporation of sulfuric acid, the excess moles of

H_2SO_4 evaporate from the solution resulting in an equimolar ionic liquid containing just HSO_4^- anions and the positively charged N-containing organic.

Additionally, we tested the ionic liquid formation with the smallest volume that we could obtain FTIR spectra for, on order of 200 nano liters (nL). We used 300 µg of glycine mixed with 20 µL of concentrated sulfuric acid (98% by weight in water) and evaporated the excess sulfuric acid using our custom evaporator. The FTIR spectra post-evaporation matches the [glycine$^+$][HSO_4^-] ionic liquid (Figure S16).

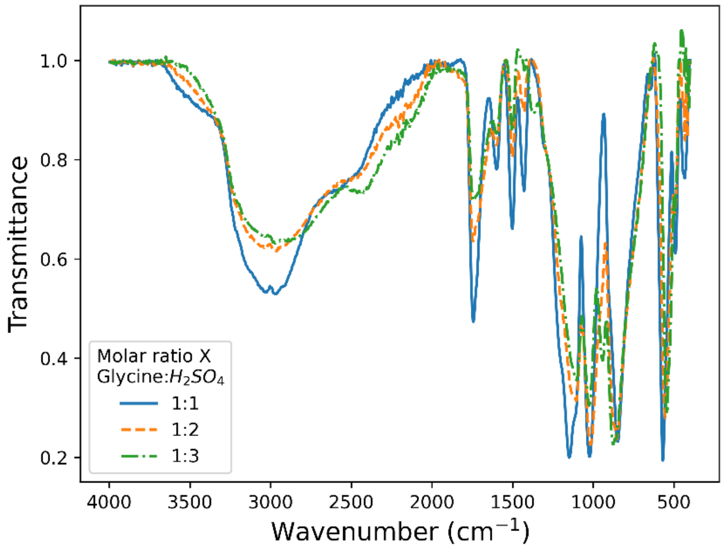

Figure S14. FTIR spectra of glycine in concentrated sulfuric acid (98% H_2SO_4 by weight, the rest water) at three molar ratios, 1:1, 1:2, and 1:3. The y-axis is transmittance and the x-axis: wavenumber in cm^{-1}. The IR spectra of the equimolar [glycine$^+$][HSO_4^-] ionic liquid mixture (blue) is distinct from the 1:2 (orange) and 1:3 (green) spectra allowing for differentiation between equimolar and non-equimolar 1:2 and 1:3, ionic liquids. The FTIR of the 1:2 and 1:3 ionic liquids have a wide O-H stretch in the 3200-2300 cm^{-1} region, whereas the 1:1 variant has a narrower O-H region 2500-3200 cm^{-1} and is shifted as compared to its 1:2 and 1:3 counterparts.

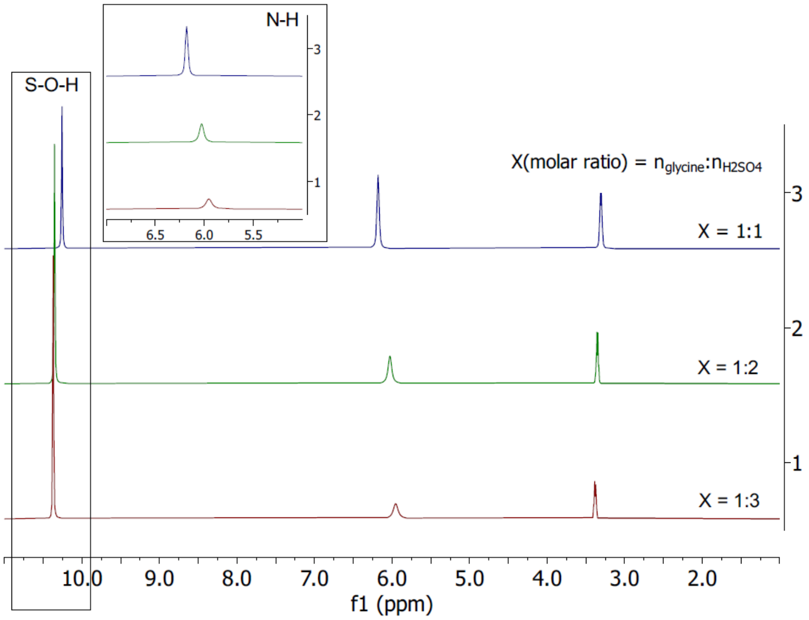

Figure S15. ^{1}H NMR spectra of glycine in concentrated sulfuric acid (98% H_2SO_4 w/w, the rest water) at three molar ratios. The spectra are recorded at 80 °C and 500.18 MHz with DMSO-d_6 as an external lock solvent. The molar composition of [glycine$^+$][HSO_4^-] ionic liquid variants are: 1:1 (blue spectra), 1:2 (green spectra), and 1:3 (red spectra). The ^{1}H NMR spectra of [glycine$^+$][HSO_4^-] ionic liquid produced by evaporation of the excess acid is consistent with an equimolar ratio ionic liquid and differs from the spectra of non-equimolar ionic liquids (1:2 and 1:3). The SO-H proton peak at 10.26 ppm is slightly shifted upfield in the 1:1 variant as compared to non-equimolar ionic liquids (1:2 and 1:3). Such an upfield shift agrees with previously reported spectra of hydrogen sulfate ionic liquids (8). Likewise, the peak corresponding to the amine protons of the glycine group, at 6.18 ppm, is slightly shifted downfield in the equimolar [glycine$^+$][HSO_4^-] ionic liquid, as compared to its 1:2 and 1:3 counterparts.

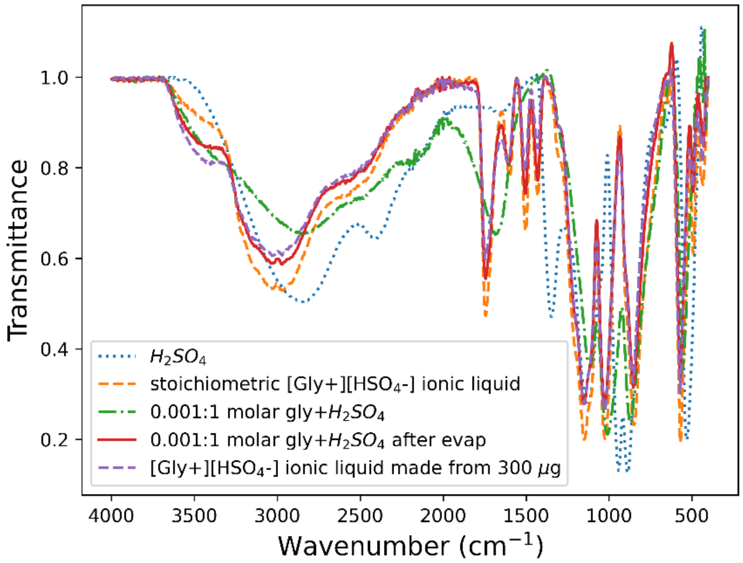

Figure S16. FTIR spectra of 20 mM glycine dissolved in 98% w/w H_2SO_4 (the rest water) before and after evaporation of excess sulfuric acid, compared to the equimolar [glycine$^+$][HSO_4^-] ionic liquid. The y-axis is transmittance and the x-axis is wavenumber in cm^{-1}. The scale of preparation, i.e. the amount of dissolved glycine, does not affect the formation and composition of the ionic liquid. The spectra of equimolar [glycine$^+$][HSO_4^-] ionic liquid look virtually identical to the spectra of ionic liquid formed from evaporated diluted solution of glycine (orange spectra, 20 mM, 0.001:1 molar ratio).

Formation of Ionic Liquids on a Basalt Rock Surface

We show that ionic liquids form outside of pure laboratory conditions, on the surface of basaltic rocks, in a variety of conditions—as long as the excess H_2SO_4 is removed. In our experiments, the acid is removed by spreading through rock pores, reacting with the rock, or evaporation. As a relevant side note, concentrated sulfuric acid itself does not readily react with basalt rock surfaces under dessicated conditions (Figure S17). Also, glycine powder deposited on the surface of basaltic rocks readily dissolves in hot liquid concentrated sulfuric acid.

In all of the experiments we used grayish black trap rock from Ward's Science company. We placed two basaltic rocks in our vacuum chamber, with the glycine-H_2SO_4 mixture on one rock and only liquid H_2SO_4 on the second rock (as a control). The hot plate was set to 120 °C, which resulted in 80 °C (+/- 5 °C) temperature of the rocks (measured with an infrared thermometer). After 24 hours, we observed that the rock with glycine was coated with liquid, whereas the control rock was almost dry, showing that excess sulfuric acid evaporated or was absorbed by the rock. The ionic liquid that formed on the basaltic rock was stable to evaporation and further reactivity with the rock surface. We note that the vacuum chamber conditions were the same those used for ionic liquid production in the glass vials (see SI Section S2 and results in Figure S18, S19 and Figure 2 in the main text);

Additionally, we show that in the presence of N-containing organics, sulfuric acid forms ionic liquid before reacting with the basaltic rock—even under open, room-temperature, room-pressure conditions including in the presence of water. We tested the formation of ionic liquid on the basaltic rock without our custom evaporator under three conditions. In the first two conditions, glycine (28 mg) was transferred to drilled cavities in the rocks and 40 µL of 98% w/w H_2SO_4 (the rest water) was added. The first rock was placed in the open lab conditions (Figure S18a) and the second rock was placed inside a desiccated container (Figure S18b). In the third condition, glycine (28 mg) was mixed with 20 µL of water and 20 µL of 98% w/w H_2SO_4 (the rest water) in the rock cavity and placed inside the desiccated container (Figure S18 c). In all three cases, there was visible liquid inside the cavities after 40 hours and the FTIR spectra of the samples matched that of the [glycine$^+$][HSO_4^-] ionic liquid prepared in glass vials (Figure S19).

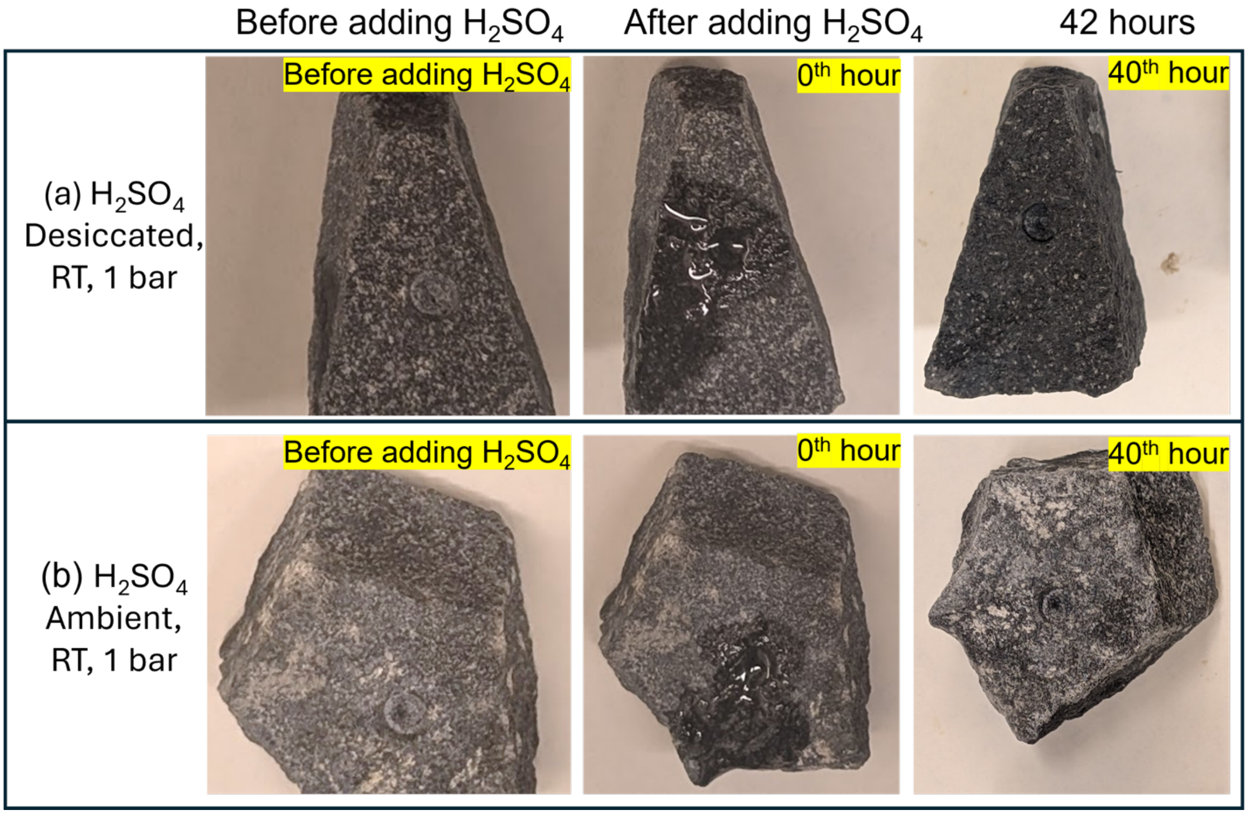

Figure S17. Concentrated sulfuric acid does not react with basalt in dry air. Top row: Basalt with 98% w/w H_2SO_4 (the rest water) stored in a desiccated jar to prevent interaction with water in the air. There is no apparent visible reaction of concentrated sulfuric acid (CSA) with the basalt rock in a desiccated environment. The rock remains coated in liquid. Bottom row: Basalt with 98% w/w H_2SO_4 (the rest water), stored in open lab conditions at all times. Water vapor from the air dilutes the acid and leads to reactivity of the diluted acid with the rock surface. Such weathering of rocks by aqueous, diluted, sulfuric acid solutions is well known (e.g., (12, 13)).

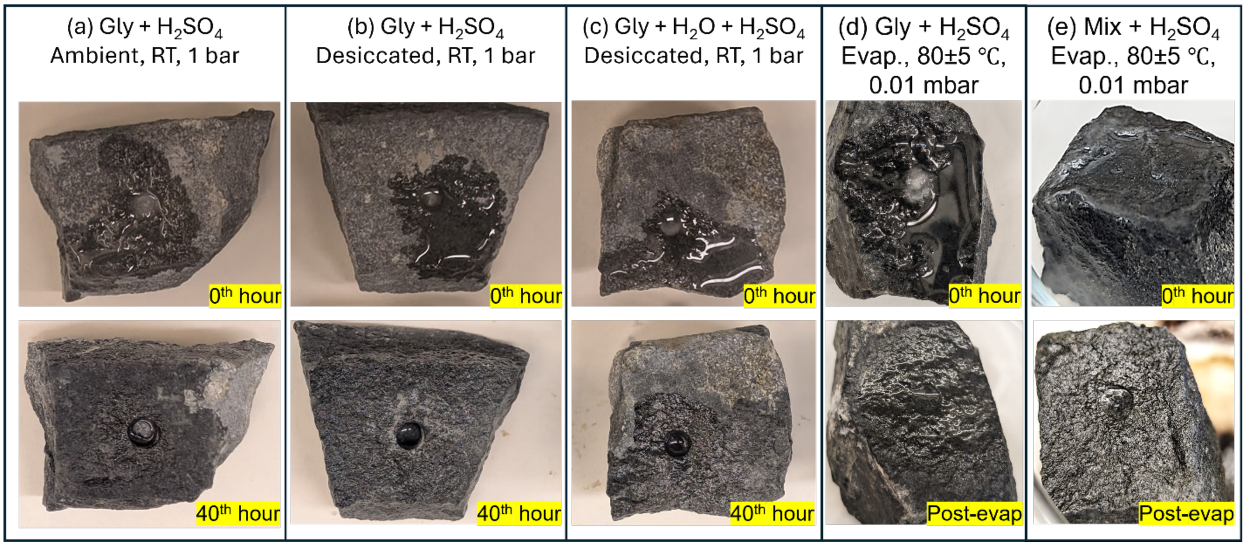

Figure S18. N-containing organics and H_2SO_4 on basalt form ionic liquids. Mix in (e) refers to a mixture of N-containing organic compounds—glycine, histidine, thymine, choline, tetrabutylammonium, and palmitic acid. Top row: 98% w/w H_2SO_4 (the rest water) and organic compounds right after mixing, with excess H_2SO_4. Bottom row: After 40 hours (a, b, c) or post evaporation (d, e). The

bottom-row images show that ionic liquids form on rock surfaces after the excess H_2SO_4 is removed—either by spreading through rock pores, reacting with the rock, or evaporation.

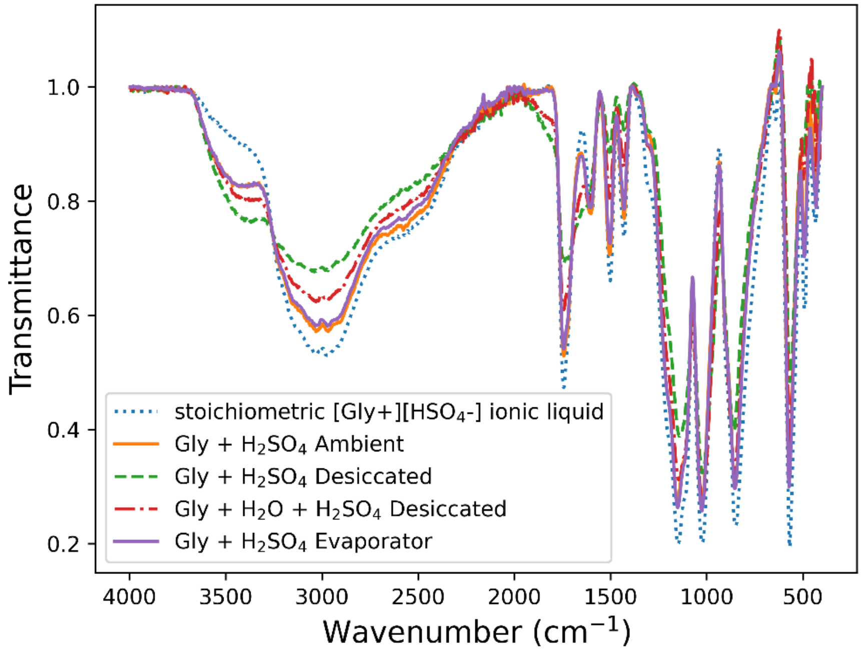

Figure S19. FTIR spectrum of [glycine$^+$][HSO_4^-] ionic liquid (blue) compared with the ionic liquid generated from a glycine-H_2SO_4 mixture on the basalt rock under various conditions. The y-axis is transmittance and the x-axis is wavenumber in cm^{-1}. The spectra match, indicating that the liquid that forms on the surface of basaltic rock is [glycine$^+$][HSO_4^-] ionic liquid. All the ionic liquid spectra look virtually identical, confirming the formation of [glycine$^+$][HSO_4^-] ionic liquid on the surface of basaltic rock from different starting conditions. Note the y-axis offsets are due to differing volumes of liquid on the FTIR measuring crystal. The shoulder at ~3500 cm^{-1} corresponds to the $\nu_{O\text{-}H}$ in H_2O and is likely due to moisture absorbed from air, due to the highly hygroscopic nature of ionic liquids.

Formation Pathways of Concentrated Sulfuric Acid

We capture an overview of concentrated sulfuric acid formation pathways on Earth, Venus, and Europa in Table S8.

Table S8. An overview of known planetary H_2SO_4 formation pathways.

**H_2SO_4 Formation Process**	**Location**	**Comments**
UV-driven oxidation of SO_2, gas phase oxidation by OH (Earth) (14–17) or O (Venus) radicals (18–20).	Atmosphere: Venus, Earth	Formation of sulfuric acid clouds on Venus and Earth stratospheric aerosols.
Atmospheric oxidation of reduced organic sulfur compounds (21).	Atmosphere: Earth	Direct production (SO_2-independent) of sulfuric acid by atmospheric oxidation of reduced sulfur-containing organic compounds.
Oxidation of SO_2 in water cloud droplets (22, 23).	Atmosphere: Earth	Aqueous phase oxidation in cloud or fog droplets by dissolved O_3, H_2O_2, NO_2, and transition metal ions.
Volcanically produced in an oxidizing environment (24).	Surface: Earth	Sulfuric acid can be directly released by volcanoes in an oxidizing environment.

Radiolysis and oxidation of surface sulfur deposits (25) (reviewed in (26)).	Surface: Europa	Sulfuric acid on the surface of Jupiter's moon Europa forms via radiolytic oxidation of sulfur deposits on Europa's icy shell.
Fe_2O_3-catalyzed oxidation of volcanically released SO_2 (27).	Surface: Earth	On Earth basaltic or mafic surface rocks containing Fe_2O_3 can catalyze SO_2 oxidation to H_2SO_4 in volcanically active environments rich in SO_2.
Heterogeneous oxidation of atmospheric SO_2 on the surfaces of various mineral dust particles: gibbsite (28), MnO_x: (29, 30), Fe_2O_3: (31, 32).	Atmosphere: Earth	Mineral dust in Earth's atmosphere can catalyze the formation of sulfuric acid.

Sulfuric Acid Evaporation Time Estimates

We provide experimentally measured timescales (Table S9) for concentrated sulfuric acid evaporation for temperatures and pressures within the liquid portion of the sulfuric acid phase diagram (Figure 5 in the main manuscript). The point is that sulfuric acid can evaporate in reasonable times for a range of planetary temperature and pressure surface conditions, in order to form ionic liquids after dissolving organics.

Table S9. Time in months to evaporate 1 mL/cm^2 of pure concentrated sulfuric acid (98% w/w concentrated sulfuric acid, the rest water) at a range of temperatures and pressures. Values are scaled from evaporation measurements shown in the four right-most columns.

**Temperature (K)**	**Pressure (atm)**	**Time to Evaporate 1 mL/cm^2 (months)**	**Volume (μL)**	**Area (mm^2)**	**Time (min)**	**Quantity Spread Over Area (mL/cm^2)**
323	1×10^{-5}	1.3	1	52	108	0.001913
363	1×10^{-3}	0.4	1	59	30	0.001682
353	4×10^{-5}	0.1	1	19	31	0.005300
343	6×10^{-5}	1.0	2	55	163	0.003661
353	7×10^{-4}	0.7	2	36	160	0.005546
323	7×10^{-4}	1.9	2	65	255	0.003070
373	1.6×10^{-3}	0.2	2	34	45	0.005961
373	1×10^{-5}	0.3	2	24	90	0.008285

Organics in the Solar System

We provide an overview of the known distribution of the organic material in the solar system in Table S10.

Table S10. Organic material in the solar system.

Planetary Body	Organics Detected	Hypothesized Mechanism of Formation	Relevance to the Planet with Surface Ionic Liquids
Mercury	Potentially large abundance of complex organics of unknown composition (33).	Unknown, possibly originally delivered to Mercury's surface by comets.	Provides a precedent for localized abundance of complex organics, possibly as thick as 10 cm, on the surface of atmosphereless bodies. The composition of the organic deposits is unknown but it is likely a complex macromolecular material formed as a result of processing of simple organic molecules into dark complex organics by high-energy photons and particles.
Luna (the Moon)	Low abundance of diverse organic molecules in lunar regolith (34–36).	Combination of meteoritic or cometary infall to the lunar surface and terrestrial contamination from the Apollo program.	N/A
Mars	Aromatic and aliphatic Cl-containing organics, S-containing organics including thiophene, decane, undecane and dodecane (the last three are pyrolytic products of carboxylic acids) (37–43).	Unknown, possibly involving past aqueous environment.	N/A
Asteroids and meteoritic material	A variety of organic molecules including amino acids, nucleic acid bases, carboxylic acids, amines, aromatic hydrocarbons (44–46).	Unknown, possibly involving past aqueous environment.	Relevant as a potential source of N-containing organic material on the planetary surface. Organic material can be highly localized and concentrated in the form of "nanoglobules".
Comets	High abundance of organics (47). Diverse organic molecules, including N-containing such as glycine, methylamine, ethylamine (48).	Possibly formed from interstellar materials that have been processed in the protosolar nebulae.	Relevant as a potential source of N-containing organic material on the planetary surface. Organic material can be highly concentrated in comet dust particles.
Ceres	Large abundance of aliphatic organics (49–52).	Formed endogenously via an unknown process potentially involving past aqueous	Provides a precedent for large abundance of endogenously produced organic molecules (if the

		environment or delivered locally by organic-rich impactors.	hypothesis on endogenous source of organics is confirmed).
Jupiter	Ethane, acetylene, other hydrocarbons (53).	Photochemically generated from methane in the atmosphere.	N/A
Ganymede	Various hydrocarbons and possibly aliphatic aldehydes (54).	Endogenous origin, inside Ganymede, possibly hydrothermal.	N/A
Callisto	Tholins and other organic molecules containing nitrile groups (55).	UV and particle radiation from the Jovian magnetosphere.	UV- and radiation-driven synthesis of organics could also proceed on the surface of an airless planet.
Titan	Large abundance of hydrocarbons and N-rich organic compounds present both as atmospheric hazes and surface deposits (56); Potential presence of large abundance of complex organics in Titan's interior (57, 58).	Photochemistry (solar UV) and atmospheric chemistry converting CH_4 and N_2 into complex organics; Unknown processes in the interior of Titan, if the interior contains large deposits of complex organics.	If large deposits of diverse organic material in the interior of Titan is confirmed, it suggests unknown endogenous processes that lead to the formation of organics within interiors of planetary bodies.
Enceladus	Complex organics (>200 atomic mass units) with diverse functional groups, including aromatics, carbonyls and N-containing molecules (59, 60).	Unknown. Possibly hydrothermal activity in the moon's interior, at the liquid water-rock interface.	N/A
Pluto and Charon	Possible presence of hydrocarbons and nitriles that are converted to complex organic molecules on the surface of the dwarf planet (61–64).	Surface ice interactions with solar UV or cosmic rays.	Potential for generation of complex N-containing organics on the surface of the planet via cosmic radiation.

S5. List of Abbreviations

Abbreviations

FTIR spectroscopy: Fourier Transform Infrared Spectroscopy
NMR spectroscopy: Nuclear Magnetic Resonance Spectroscopy
TGA: Thermogravimetric Analysis

Chemical Nomenclature

The cation names for amino acids (e.g., glycininium, alaninium) reflect their protonated forms, as is standard in amino acid-based ionic liquid naming conventions. The naming of ionic liquids containing nucleic acid bases (thymine, purine, guanine) follows similar convention (e.g., thyminium). For complexes with additional H_2SO_4, the neutral molecule is included in the name.

[glycine$^+$][HSO_4^-]: Glycininium hydrogen sulfate
[glycine$^+$][HSO_4^-][H_2SO_4]: Glycininium hydrogen sulfate sulfuric acid
[glycine$^+$][HSO_4^-][H_2SO_4]$_2$: Glycininium hydrogen sulfate bis(sulfuric acid)
[histidine$^+$][HSO_4^-]: Histidinium hydrogen sulfate
[alanine$^+$][HSO_4^-]: Alaninium hydrogen sulfate
[cysteine$^+$][HSO_4^-]: Cysteinium hydrogen sulfate
[valine$^+$][HSO_4^-]: Valinium hydrogen sulfate
[thymine$^+$][HSO_4^-]: Thyminium hydrogen sulfate
[purine$^+$][HSO_4^-]: Purinium hydrogen sulfate
[guanine$^+$][HSO_4^-]: Guaninium hydrogen sulfate
[1-ethyl-3-methylimidazolium$^+$][HSO_4^-]: 1-Ethyl-3-methylimidazolium hydrogen sulfate
[tetrabutylammonium$^+$][HSO_4^-]: Tetrabutylammonium hydrogen sulfate
[alanylglycine$^+$][HSO_4^-]: Alanylglycinium hydrogen sulfate
[choline$^+$][HSO_4^-]: choline hydrogen sulfate

DMSO-d_6: dimethyl sulfoxide-d_6

S6. Supplementary Datasets

Supplementary Dataset S1: The original ^{1}H NMR, ^{31}P NMR data used in support of the chemical and physical characterization of ionic liquid formation. All data can be downloaded from Zenodo at https://zenodo.org/records/15596466.
Supplementary Dataset S2: The original FTIR data used in support of the chemical and physical characterization of ionic liquid formation. All data can be downloaded from Zenodo at https://zenodo.org/records/15596466.
Supplementary Dataset S3: The original TGA data used in support of the characterization of ionic liquid formation. All data can be downloaded from Zenodo at https://zenodo.org/records/15596466.

S7. Supplementary References

1. J. S. Wilkes, A short history of ionic liquids—from molten salts to neoteric solvents. *Green Chem.* **4**, 73–80 (2002).
2. A. V Kotov, V. A. Zarinskii, V. M. Bokina, Mechanism of the interaction of concentrated sulfuric acid and glacial acetic acid. *Bull. Acad. Sci. USSR, Div. Chem. Sci.* **18**, 1217–1219 (1969).
3. G. P. Lorenzi, V. Rizzo, F. Thoresen, L. Tomasic, Circular Dichrosim and Conformational Equilibrium of Homopoly-L-peptides with Alkyl Side Chains in Concentrated Sulfuric Acid. *Macromolecules* **12**, 870–874 (1979).
4. J. Steigman, E. Peggion, A. Cosani, Protonation of peptides. I. Behavior of a model diamide and of poly (. gamma.-ethyl-L-glutamate) in strong acid-water mixtures. *J. Am. Chem. Soc.* **91**, 1822–1829 (1969).
5. E. Peggion, A. Cosani, M. Terbojevich, A. S. Verdini, Circular Dichroism Studies on Poly-L-lysine in Water-Sulfuric Acid Mixtures. *Macromolecules* **3**, 318–322 (1970).
6. M. D. Seager, S. Seager, W. Bains, J. J. Petkowski, Stability of 20 Biogenic Amino Acids in Concentrated Sulfuric Acid: Implications for the Habitability of Venus' Clouds. *Astrobiology* **24**, 386–396 (2024).
7. J. J. Petkowski, M. D. Seager, W. Bains, J. H. Grimes Jr, S. Seager, Mechanism for Peptide Bond Solvolysis in 98% w/w Concentrated Sulfuric Acid. *ACS omega* **10**, 9623–9629 (2025).
8. K. Matuszek, A. Chrobok, F. Coleman, K. R. Seddon, M. Swadźba-Kwaśny, Tailoring ionic liquid catalysts: structure, acidity and catalytic activity of protonic ionic liquids based on anionic clusters,$[(HSO_4)(H_2SO_4)_x]^-$(x= 0, 1, or 2). *Green Chem.* **16**, 3463–3471 (2014).
9. U. Mayer, V. Gutmann, W. Gerger, The acceptor number—A quantitative empirical parameter for the electrophilic properties of solvents. *Monatshefte für Chemie/Chemical Mon.* **106**, 1235–1257 (1975).
10. M. A. Beckett, G. C. Strickland, J. R. Holland, K. S. Varma, A convenient nmr method for the measurement of Lewis acidity at boron centres: correlation of reaction rates of Lewis acid initiated epoxide polymerizations with Lewis acidity. *Polymer (Guildf).* **37**, 4629–4631 (1996).
11. J. You, *et al.*, Non-stoichiometric protic ionic liquids. *J. Mol. Liq.*, 125663 (2024).
12. E. M. Hausrath, D. C. Golden, R. V Morris, D. G. Agresti, D. W. Ming, Acid sulfate alteration of fluorapatite, basaltic glass and olivine by hydrothermal vapors and fluids: Implications for fumarolic activity and secondary phosphate phases in sulfate-rich Paso Robles soil at Gusev Crater, Mars. *J. Geophys. Res. Planets* **118**, 1–13 (2013).
13. D. C. Golden, D. W. Ming, R. V Morris, S. A. Mertzman, Laboratory-simulated acid-sulfate weathering of basaltic materials: Implications for formation of sulfates at Meridiani Planum and Gusev crater, Mars. *J. Geophys. Res. Planets* **110** (2005).
14. R. L. Mauldin Iii, *et al.*, A new atmospherically relevant oxidant of sulphur dioxide. *Nature* **488**, 193–196 (2012).
15. R. A. Cox, S. A. Penkett, Photo-oxidation of atmospheric SO_2. *Nature* **229**, 486–488 (1971).
16. R. A. Cox, Photochemical oxidation of atmospheric sulphur dioxide. *Philos. Trans. R. Soc. London. Ser. A, Math. Phys. Sci.* **290**, 543–550 (1979).
17. D. D. Davis, A. R. Ravishankara, S. Fischer, SO_2 oxidation via the hydroxyl radical: Atmospheric fate of HSOx radicals. *Geophys. Res. Lett.* **6**, 113–116 (1979).
18. V. A. Krasnopolsky, Chemical kinetic model for the lower atmosphere of Venus. *Icarus* **191**, 25–37 (2007).
19. V. A. Krasnopolsky, A photochemical model for the Venus atmosphere at 47–112 km.

Icarus **218**, 230–246 (2012).
20. P. B. Rimmer, *et al.*, Hydroxide salts in the clouds of Venus: their effect on the sulfur cycle and cloud droplet pH. *Planet. Sci. J.* **2**, 133 (2021).
21. T. Berndt, E. H. Hoffmann, A. Tilgner, F. Stratmann, H. Herrmann, Direct sulfuric acid formation from the gas-phase oxidation of reduced-sulfur compounds. *Nat. Commun.* **14**, 4849 (2023).
22. H.-M. Hung, M. R. Hoffmann, Oxidation of gas-phase SO_2 on the surfaces of acidic microdroplets: Implications for sulfate and sulfate radical anion formation in the atmospheric liquid phase. *Environ. Sci. Technol.* **49**, 13768–13776 (2015).
23. V.-M. Kerminen, *et al.*, Interaction between SO2 and submicron atmospheric particles. *Atmos. Res.* **54**, 41–57 (2000).
24. M. Zelenski, Y. Taran, B. Galle, High emission rate of sulfuric acid from Bezymianny volcano, Kamchatka. *Geophys. Res. Lett.* **42**, 7005–7013 (2015).
25. R. W. Carlson, R. E. Johnson, M. S. Anderson, Sulfuric acid on Europa and the radiolytic sulfur cycle. *Science (80-.).* **286**, 97–99 (1999).
26. K. Lodders, B. Fegley, Sulfur in the Giant Planets, their Moons, and Extrasolar Gas Giant Planets. *arXiv Prepr. arXiv2410.11138* (2024).
27. A. K. Pattantyus, S. Businger, S. G. Howell, Review of sulfur dioxide to sulfate aerosol chemistry at Kīlauea Volcano, Hawai ʻi. *Atmos. Environ.* **185**, 262–271 (2018).
28. J. Yun, C. Zhu, Q. Wang, Q. Hu, G. Yang, Catalytic conversions of atmospheric sulfur dioxide and formation of acid rain over mineral dusts: Molecular oxygen as the oxygen source. *Chemosphere* **217**, 18–25 (2019).
29. H. Wang, C. Zhong, Q. Ma, J. Ma, H. He, The adsorption and oxidation of SO_2 on MgO surface: experimental and DFT calculation studies. *Environ. Sci. Nano* **7**, 1092–1101 (2020).
30. W. Yang, *et al.*, Heterogeneous reaction of SO_2 on manganese oxides: the effect of crystal structure and relative humidity. *Sci. Rep.* **7**, 4550 (2017).
31. K. Li, *et al.*, Heterogeneous conversion of SO_2 on nano α-Fe_2O_3: the effects of morphology, light illumination and relative humidity. *Environ. Sci. Nano* **6**, 1838–1851 (2019).
32. H. Fu, X. Wang, H. Wu, Y. Yin, J. Chen, Heterogeneous uptake and oxidation of SO_2 on iron oxides. *J. Phys. Chem. C* **111**, 6077–6085 (2007).
33. D. A. Paige, *et al.*, Thermal stability of volatiles in the north polar region of Mercury. *Science (80-.).* **339**, 300–303 (2013).
34. J. E. Elsila, *et al.*, The origin of amino acids in lunar regolith samples. *Geochim. Cosmochim. Acta* **172**, 357–369 (2016).
35. K. L. Thomas-Keprta, *et al.*, Organic matter on the Earth's Moon. *Geochim. Cosmochim. Acta* **134**, 1–15 (2014).
36. J. E. Elsila, *et al.*, Soluble organic compounds and cyanide in Apollo 17 lunar samples: Origins and curation effects. *J. Geophys. Res. Planets* **129**, e2023JE008133 (2024).
37. C. Freissinet, *et al.*, Long-chain alkanes preserved in a Martian mudstone. *Proc. Natl. Acad. Sci.* **122**, e2420580122 (2025).
38. C. Szopa, *et al.*, First detections of dichlorobenzene isomers and trichloromethylpropane from organic matter indigenous to Mars mudstone in Gale Crater, Mars: results from the Sample Analysis at Mars instrument onboard the Curiosity rover. *Astrobiology* **20**, 292–306 (2020).
39. M. Millan, *et al.*, Organic molecules revealed in Mars's Bagnold Dunes by Curiosity's derivatization experiment. *Nat. Astron.* **6**, 129–140 (2022).
40. M. Millan, *et al.*, Sedimentary organics in Glen Torridon, Gale Crater, Mars: Results from the SAM instrument suite and supporting laboratory analyses. *J. Geophys. Res. Planets* **127**, e2021JE007107 (2022).

41. D. P. Glavin, *et al.*, Evidence for perchlorates and the origin of chlorinated hydrocarbons detected by SAM at the Rocknest aeolian deposit in Gale Crater. *J. Geophys. Res. Planets* **118**, 1955–1973 (2013).
42. C. Freissinet, *et al.*, Organic molecules in the sheepbed mudstone, gale crater, mars. *J. Geophys. Res. Planets* **120**, 495–514 (2015).
43. J. L. Eigenbrode, *et al.*, Organic matter preserved in 3-billion-year-old mudstones at Gale crater, Mars. *Science (80-.).* **360**, 1096–1101 (2018).
44. D. P. Glavin, *et al.*, Abundant ammonia and nitrogen-rich soluble organic matter in samples from asteroid (101955) Bennu. *Nat. Astron.*, 1–12 (2025).
45. H. Naraoka, *et al.*, Soluble organic molecules in samples of the carbonaceous asteroid (162173) Ryugu. *Science (80-.).* **379**, eabn9033 (2023).
46. H. G. Changela, *et al.*, Morphological Study of Insoluble Organic Matter Residues from Primitive in *43rd Lunar and Planetary Science Conference*, (2012).
47. A. Bardyn, *et al.*, Carbon-rich dust in comet 67P/Churyumov-Gerasimenko measured by COSIMA/Rosetta. *Mon. Not. R. Astron. Soc.* **469**, S712–S722 (2017).
48. K. Altwegg, *et al.*, Prebiotic chemicals—amino acid and phosphorus—in the coma of comet 67P/Churyumov-Gerasimenko. *Sci. Adv.* **2**, e1600285 (2016).
49. M. C. De Sanctis, *et al.*, Localized aliphatic organic material on the surface of Ceres. *Science (80-.).* **355**, 719–722 (2017).
50. H. H. Kaplan, R. E. Milliken, C. M. O. Alexander, New constraints on the abundance and composition of organic matter on Ceres. *Geophys. Res. Lett.* **45**, 5274–5282 (2018).
51. S. Marchi, *et al.*, An aqueously altered carbon-rich Ceres. *Nat. Astron.* **3**, 140–145 (2019).
52. R. Sarkar, *et al.*, Ceres: Organic-Rich Sites of Exogenic Origin? *AGU Adv.* **6**, e2024AV001362 (2025).
53. H. Melin, *et al.*, Assessing the long-term variability of acetylene and ethane in the stratosphere of Jupiter. *Icarus* **305**, 301–313 (2018).
54. F. Tosi, *et al.*, Salts and organics on Ganymede's surface observed by the JIRAM spectrometer onboard Juno. *Nat. Astron.* **8**, 82–93 (2024).
55. T. B. a McCord, *et al.*, Organics and other molecules in the surfaces of Callisto and Ganymede. *Science (80-.).* **278**, 271–275 (1997).
56. M. L. Cable, *et al.*, Titan tholins: simulating Titan organic chemistry in the Cassini-Huygens era. *Chem. Rev.* **112**, 1882–1909 (2012).
57. K. E. Miller, D. I. Foustoukos, G. D. Cody, C. M. O. Alexander, Experimental heating of complex organic matter at Titan's interior conditions supports contributions to atmospheric N2 and CH4. *Geochim. Cosmochim. Acta* **390**, 38–56 (2025).
58. K. E. Miller, C. R. Glein, J. H. Waite, Contributions from accreted organics to Titan's atmosphere: new insights from cometary and chondritic data. *Astrophys. J.* **871**, 59 (2019).
59. F. Postberg, *et al.*, Macromolecular organic compounds from the depths of Enceladus. *Nature* **558**, 564–568 (2018).
60. N. Khawaja, *et al.*, Low-mass nitrogen-, oxygen-bearing, and aromatic compounds in Enceladean ice grains. *Mon. Not. R. Astron. Soc.* **489**, 5231–5243 (2019).
61. M. Fayolle, *et al.*, Testing tholins as analogues of the dark reddish material covering Pluto's Cthulhu region. *Icarus* **367**, 114574 (2021).
62. M. L. Wong, *et al.*, The photochemistry of Pluto's atmosphere as illuminated by New Horizons. *Icarus* **287**, 110–115 (2017).
63. P. Gao, *et al.*, Constraints on the microphysics of Pluto's photochemical haze from New Horizons observations. *Icarus* **287**, 116–123 (2017).
64. E. Lellouch, *et al.*, Detection of CO and HCN in Pluto's atmosphere with ALMA. *Icarus* **286**, 289–307 (2017).
